# The DISC1 Pathway Modulates Expression of Neurodevelopmental, Synaptogenic and Sensory Perception Genes

**DOI:** 10.1371/journal.pone.0004906

**Published:** 2009-03-20

**Authors:** William Hennah, David Porteous

**Affiliations:** Medical Genetics Section, University of Edinburgh, Edinburgh, United Kingdom; University of Wuerzburg, Germany

## Abstract

**Background:**

Genetic and biological evidence supports a role for DISC1 across a spectrum of major mental illnesses, including schizophrenia and bipolar disorder. There is evidence for genetic interplay between variants in DISC1 and in biologically interacting loci in psychiatric illness. DISC1 also associates with normal variance in behavioral and brain imaging phenotypes.

**Methodology:**

Here, we analyze public domain datasets and demonstrate correlations between variants in the DISC1 pathway genes and levels of gene expression. Genetic variants of DISC1, NDE1, PDE4B and PDE4D regulate the expression of cytoskeletal, synaptogenic, neurodevelopmental and sensory perception proteins. Interestingly, these regulated genes include existing targets for drug development in depression and psychosis.

**Conclusions:**

Our systematic analysis provides further evidence for the relevance of the DISC1 pathway to major mental illness, identifies additional potential targets for therapeutic intervention and establishes a general strategy to mine public datasets for insights into disease pathways.

## Introduction

A key challenge for human genomics is to provide insight into normal physiological processes and pathogenic mechanisms. There is strong evidence for a major genetic component to schizophrenia and bipolar disorder, but, with some notable exceptions, few of the many proposed candidate genes have been consistently replicated [1], [2]. It has been argued that much of the genetic variance associated with common complex disease and quantitative trait variance is likely to be regulatory rather than coding [3]. The combination of high density SNP analysis with expression profiling provides a means to assess the genome wide control of gene expression [4]. This approach has been applied successfully to the analysis of EBV transformed lymphoblastoid cell lines and of human tissue [4]. Here, we apply the approach specifically to the DISC1 pathway for additional insight into genetic mediators of psychosis and related biology.

Studies of DISC1 and its interactors have established this as one of the most promising pathways underlying psychosis. This evidence includes a) linkage and association signals across the DISC locus for multiple psychiatric, cognitive and brain imaging [5] traits, b) binding of DISC1 to multiple protein partners with known roles in neurobiology [5] and c) mouse models of Disc1 by ENU missense mutagenesis [6] or transgenic over-expression [7]–[9], which display overlapping neurodevelopmental and behavioral phenotypes, with abnormal working memory as a core shared deficit. Of direct relevance to this study is the recent evidence for genetic interplay between DISC1 variants [10] and observations of association to psychiatric illness for the DISC1 interactors NDE1, NDEL1, PDE4B, and PDE4D [11], [12](Text S1). Functional studies of these proteins have shown that they are involved in cytoskeletal, and nervous system development related functions, including synaptic plasticity and neuronal migration [5]. Although psychosis is a brain disorder, DISC1, NDE1, NDEL1, PDE4B and PDE4D and many other members of the known DISC1 interactome are expressed in lymphoblastoid cell lines. We conjectured that a global analysis of normal variance in gene expression in lymphoblastoid cells might provide useful self-standing insights into pathway biology and complement clinically and technically challenging limitations of comparative post-mortem brain expression studies [13]. We have therefore mined publicly available expression data derived from lymphoblastoid cell lines of HapMap individuals for significant alterations in genome wide gene expression levels, mediated by genetic variations in the DISC1 pathway. We confirm the involvement of DISC1, NDE1, PDE4B and PDE4D in cytoskeletal, synaptogenic and neurodevelopmental functions, and provide new evidence that this pathway may also play a role in sensory perception.

## Results

**Figure 1:**
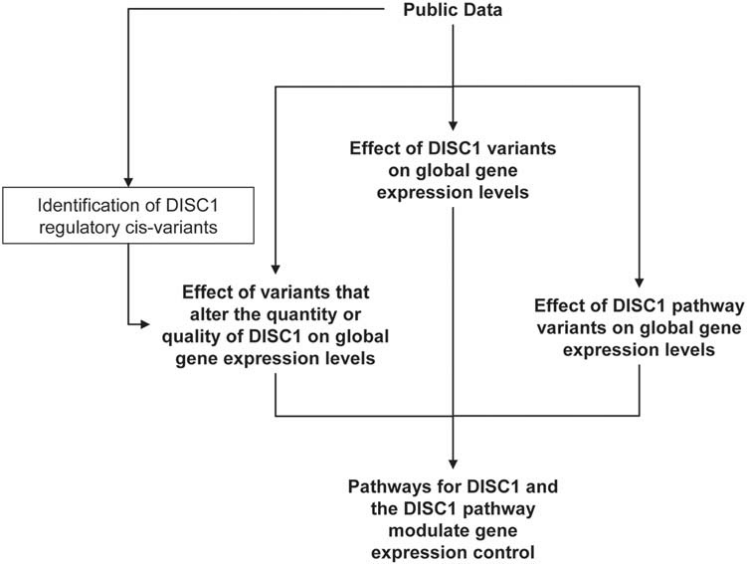
Flow chart to demonstrate the analysis performed here. This protocol can be adapted to any chosen gene, pathway and disorder by simply replacing DISC1 with the gene of primary interest.

Previous studies have shown that expression levels of full length DISC1 are reduced by half in lymphoblastoid cell lines derived from t(1;11) cases [14] and that S704C missense variants alter binding of NDEL1 [15], arguing that altering either the quality or the quantity of DISC1 can be pathognomonic But, as yet, there have been no studies to determine whether the cellular effects of these alternative genetic mechanisms are fundamentally similar or distinct. To address this, we devised a data mining and integration strategy, summarized in Figure 1. First, we examined the effect of a) six novel variants shown here to exert an effect in cis on DISC1 expression b) three common missense variants R264Q (rs3738401), L607F (rs6675281) and S704C (rs821616), c) the 3 SNPs, rs1538979, rs821577 and rs821633, reported to show interplay conferring ‘risk’, ‘neutral’ and ‘protective’ effects on schizophrenia and bipolar disorder [10] and d) variants previously reported as associated with schizophrenia or related psychotic traits in European cohorts for the DISC1 interactors NDE1, NDEL1, PDE4B and PDE4D.

Novel DISC1 cis-acting variants were identified by testing SNPs from 10 kb upstream of the immediately adjacent gene TSNAX to 10 kb downstream of DISC1 for association to expression values of DISC1. Expression values were from four publicly available data sets (GSE6536 in NCBI GEO) drawn from a common set of 210 lymphoblastoid cell lines from the four HapMap population cohorts; 60 CEU, 60 YRI, 45 CHB and 45 JPT [16]. Of the 754 variants tested, only one, rs1765778, located 30 kb upstream of DISC1, displayed significant association in all four populations (p-value range  = 0.049−0.0094). A further 5 SNPs displayed significant association (p<0.05) in three of the four populations, the common exception being the Japanese population. One of these five SNPs, rs3738398 is located 221 bp upstream of the DISC1 start site and 75 bp upstream of the CpG island that spans exon 1 of DISC1, suggesting that these variants may be representing the as yet undefined promoter region of DISC1. All six variants locate within the same CEU D′ haplotype block, which is also the longest haplotype block of the four populations (Figure S1 and Table S1), and have an average r^2^ between them of 0.69, and a maximum r^2^ of 0.87 between rs1765778 and rs1655297. In all six instances, the minor alleles were associated with decreased DISC1 expression. Carriers of the minor alleles in the CEU cohort have, on average, a reduction of 14.81% (95% CI = 13.96–15.66) in DISC1 expression, whilst homozygotes of the minor alleles have on average a 18.97% (95% CI = 17.84–20.10) reduction.

We then tested by ANOVA the genotypes of the three missense polymorphisms, the three genetic interplay variants (comparing ‘risk’, ‘neutral’ and ‘protective’ genotypes), and the six cis-acting variants for global effects on gene expression (Table S2). This used public datasets from three studies that used the CEU population [16]–[18], with each study using a different sub-cohort of individuals and different platform to measure expression levels. Only those genes that were significantly differentially expressed (p<0.05) and in the same direction across all array test platforms were considered for gene ontology analysis (materials and methods & Table S3). To test for shared effects down-stream of DISC1, the same global analysis was performed on other members of the DISC1 pathway for which variants had previously been reported as associated with schizophrenia or related psychotic traits in European cohorts. These comprised: for NDE1, 4 SNPs and 1 haplotype; for NDEL1, 1 SNP and 1 haplotype; for PDE4B, 3 SNPs and 2 haplotypes; and for PDE4D, 1 SNP and 1 haplotype [11], [12](Text S1 and Table S2).

Of the total 77154 gene targets represented by all three platforms, 13145 genes are targeted by more than one experiment. Using the 5% level, we expected 25.71 changes per variant tested in the cross-sample analysis performed (Table S3). Greater than expected numbers of genes affected were seen for eight of the fourteen variants tested, three of them significantly greater at the 0.05 level. These were DISC1 rs3738401 (R264Q) (n = 47, p = 0.012); NDE1 SNPs rs4781678 (n = 39), rs2242549 (n = 29), rs881803 (n = 52, p = 0.0028); the NDE1 tagging haplotype (n = 36); the PDE4B SNP rs7412571 (n = 83, p = 0.000000037) and haplotype (n = 31); and the PDE4D haplotype (n = 36) (Table S3).

**Figure 2:**
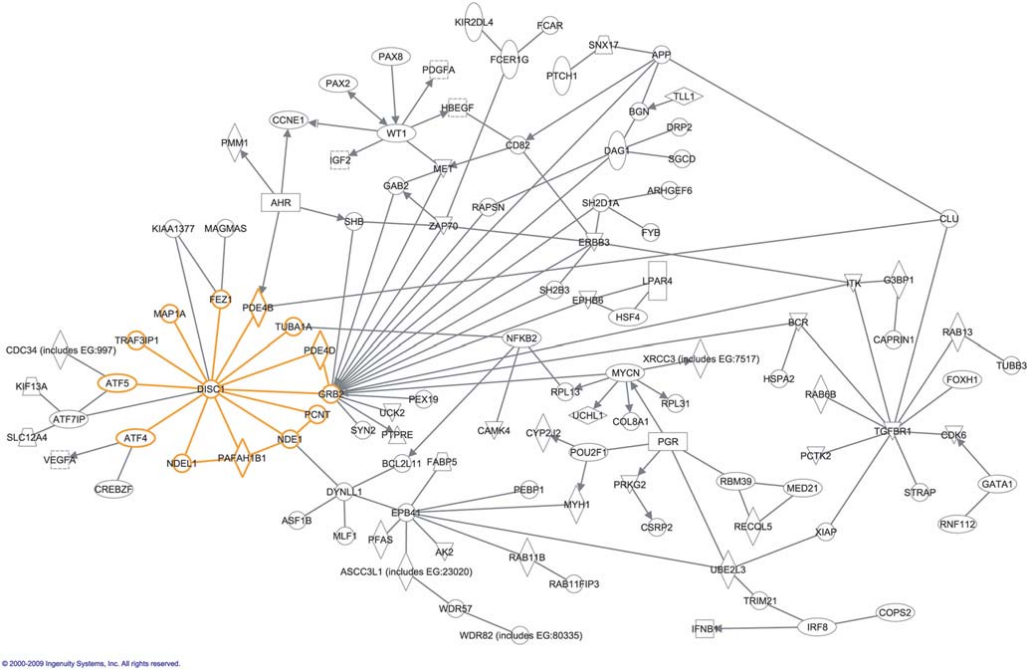
Network of how 100 of the 528 genes identified with significant differential expression relate to DISC1and its core interactors. Network generated through the use of Ingenuity Pathway Analysis (Ingenuity Systems®, www.ingenuity.com), using the build function and connecting the identified genes to the core DISC1 interactome, only allowing for direct interaction. Relationships are supported by at least 1 reference from the literature, from a textbook, or from canonical information stored in the Ingenuity knowledge base.

528 genes (4.02% of all targets represented on multiple array platforms) were differentially expressed across all variants tested (Table S3). Of these, 17 genes (3.21%) were influenced by variants in more than one DISC1 pathway gene. Of the total 178 genes identified by all DISC1 variants, 11 (6.17%) were also identified by variants in the other DISC1 pathway genes. Expression of TUBB3 was uniquely affected by both regulatory and missense DISC1 variants. 35 of the 528 genes (6.63%) have pre-existing supporting evidence for a role in psychosis. Seven genes, ATF7IP, BCR, CLU, DYNLL1, FEZ1, SNAP91 and SYN2, are interactors of DISC1 or the extended DISC1 pathway [19]. Seven genes, APP [20], ERBB3 [20], FEZ1 [21], HSPA2 [20], SERPINI1 [20], SOX10 [21] and SYN2 [22], display altered expression in studies of schizophrenia or bipolar disorder. Six genes, CLOCK [23], FEZ1, GRIA3, NOTCH4, SYN2, and TACR1, have been reported associated with schizophrenia (www.schizophreniaforum.org/res/sczgene/default.asp; [2]) or bipolar disorder. Fifteen genes, ALKBH4 [24], BCR [25], CD160 [26], DAZ3 [24], GJA5 [24], [25], IL9R [24], KIF13A [26], MGLL [25], NLGN4Y [24], PRKY [24], RGS12 [26], SCHIP1 [25], USP7 [25], VCY [24] and ZNF140 [25], are subject to copy number variation in individuals with schizophrenia. Further, the Ingenuity Pathway Analysis (Ingenuity® Systems, www.ingenuity.com) database identifies 139 genes as being targets for drugs for psychiatric illness. Of these 139, the expression of seven genes (5%), namely CA12, CHRNA5, GRIA3, HTR3A, SCN9A, TUBB3 and TUBD1 were altered by the presence of DISC1 pathway variants (number expected by chance to be observed on multiple platforms and be a known drug target = 0.29; p = 0.0074 (Table 1). Only the DISC1 variant rs3738401 (R264Q) affected more than one of these target genes, significantly altering the expression levels of CHRNA5, TUBB3 and TUBD1. Ingenuity Pathway Analysis (Ingenuity® Systems, www.ingenuity.com) also enabled additional exploration of how the identified genes relate to DISC1 and the DISC1 pathway. Using the build function and connecting the 528 genes to the core DISC1 interactome, only allowing for direct interaction, 100 (18.94%) genes can be generated into a network (Figure 2) with relationships supported by at least 1 reference from the literature, from a textbook, or from canonical information stored in the Ingenuity knowledge base.

**Table 1:** Psychiatric drug target genes that display DISC1 pathway mediated differential expression.

Target	Gene function	Mediating Variant	Treated Disorder	Drug	Action
CA12	Zinc metalloenzyme	NDE1 Haplotype	Bipolar Disorder	Topiramate	Inhibitor
SCN9A	Sodium channel subunit	PDE4B (rs7412571)	Bipolar Disorder and Depression	Riluzole	Blocker
GRIA3	Glutamate receptor	NDE1 (rs2242549)	Depression	ORG24448	Modulator
HTR3A	Serotonin receptor	NDE1 (rs4781678)	Schizophrenia	Memantine	Antagonist
			Depression and Anxiety	Mirtazapine	Antagonist
CHRNA5	Cholinergic receptor	DISC1 (rs823167, rs3738401, rs821616)	Psychosis and ADHD	ABT-089	Agonist
TUBB3	Neural microtubule dynamics	DISC1 (rs1025526, rs6541280, rs3738401)	Schizophrenia	AL 108	Binder
TUBD1	Centrosomal	DISC1 (rs1025526, rs6541280, rs3738401)	Schizophrenia	AL 108	Binder

To determine whether these 10 DISC1 and 14 DISC1 pathway variants identified convergent biological pathways, the gene ontology (GO) tree machine [27] was used to test for over abundance of similar functions (Table S4). GO term searches were performed on gene sets identified by a) individual variants that exceeded the expectation level, b) multiple variants in linkage disequilibrium with each other (that is all DISC1 ‘cis’ variants, and all NDE1 variants) and c) variants in different DISC1 pathway genes (all major mental illness associated variants, and all variants). Significant enrichment was observed for: cytoskeletal functions (DISC1, rs3738401 (p-value range, 0.0047−0.00039) and NDE1, haplotype (p-value range, 0.0060−0.0010)), synaptogenesis (PDE4B, rs7412571 (p-value = 0.0035)), transcription factor activity (DISC1, rs3738401 (p-value = 0.010) PDE4B rs7412571 (p-value range, 0.0053−0.0048)) and sensory perception (NDE1, rs881803 (p-value = 0.0038). Cytoskeletal functions and synaptogenesis have already been identified as functions of the DISC1 pathway [5]. Cytoskeletal functions were also highlighted with DISC1 ‘cis’ variants, ‘all DISC1 pathway variants’ and ‘all associated variants’. Nervous system development is also a predicted function of the DISC1 pathway [5] and was affected here by the PDE4B haplotype comprising rs2503166, rs583018 and rs526772 (p-value = 0.0014). Additionally, the gene lists for the DISC1 ‘cis’ variants, rs6541280 and rs823167, were also enriched for sensory perception gene ontology terms despite affecting fewer genes than would be expected by chance. When genes identified by associating variants in more than one DISC1 pathway member were considered, there was a significant over representation of genes involved in cytoskeleton organization and biogenesis. When all DISC1 missense and regulatory variants were added to the analysis, GO term enrichment was observed for neurotransmitter receptor activity and in G-protein coupled peptide receptor activity (Table 2, Figure S2 and Table S4). Our analysis also supports previous suggestions [10], [28] that mutations affecting either the quality or quantity of DISC1 may have subtly different phenotypic and pathogenic consequences.

**Table 2:** Table of significant gene ontology terms that are biologically relevant to the DISC1 pathway or major mental illness.

Variant	Gene Number	Gene Ontology Term	p-value	Proteins Involved
DISC1 rs1025526	19	peptide receptor activity	0.0091	TUBB3; FPR1
		peptide receptor activity\, G-protein coupled	0.0091	TUBB3; FPR1
DISC1 rs6541280	19	sensory perception of mechanical stimulus	0.0093	WFS1; CD151
		sensory perception of sound	0.0093	WFS1; CD151
DISC1 rs823167	19	sensory perception of mechanical stimulus	0.0093	WFS1; CD151
		sensory perception of sound	0.0093	WFS1; CD151
DISC1 rs3738401	47	microtubule organizing center	0.00077	PPP4R2; MLF1; TUBD1
		centrosome	0.00049	PPP4R2; MLF1; TUBD1
		microtubule cytoskeleton	0.0049	TUBB3; PPP4R2; MLF1; TUBD1
NDE1 Haplotype	36	actin cytoskeleton organization and biogenesis	0.0076	AVIL; EPB41; ARHGAP6
		actin cytoskeleton	0.0027	AVIL; DAG1; EPB41; ARHGAP6
NDE1 rs881803	52	sensory perception of mechanical stimulus	0.0085	TRIOBP; GJB3; SOX10
		sensory perception of sound	0.0085	TRIOBP; GJB3; SOX10
PDE4B Haplotype*	4	system development	0.0025	DRP2; GAS7
		nervous system development	0.0024	DRP2; GAS7
PDE4B rs7412571	83	synapse organization and biogenesis	0.0067	PCDHB13; PCDHB2
		synaptogenesis	0.0067	PCDHB13; PCDHB2
DISC1 cis	20	sensory perception of light stimulus	0.0021	OCLM; WFS1; CD151
		visual perception	0.0021	OCLM; WFS1; CD151
Associated Variants	5	organelle organization and biogenesis	0.0051	LIMA1; SMC1A
		cytoskeleton organization and biogenesis	0.0010	LIMA1; SMC1A
All Variants	17	organelle organization and biogenesis	0.0057	TUBB3; TIMM8A; LIMA1; SMC1A
		cytoskeleton organization and biogenesis	0.0044	TUBB3; LIMA1; SMC1A
		microtubule-based process	0.0059	TUBB3; SMC1A
		neurotransmitter binding	0.0045	CHRNA5; TACR1
		neurotransmitter receptor activity	0.0041	CHRNA5; TACR1
		peptide receptor activity	0.0065	TUBB3; TACR1
		peptide receptor activity\, G-protein coupled	0.0065	TUBB3; TACR1
		transmembrane receptor activity	0.0064	TUBB3; CHRNA5; P2RY10; TACR1
		G-protein coupled receptor activity	0.0068	TUBB3; P2RY10; TACR1
		rhodopsin-like receptor activity	0.0037	TUBB3; P2RY10; TACR1
		cytoskeletal part	0.0081	TUBB3; PPP4R2; LIMA1

* Haplotype of SNPs rs2503166, rs583018 and rs526772.

## Discussion

Our study has highlighted the role of six DISC1 cis-variants in significantly altering the expression levels of the DISC1 gene in a semi-dominant fashion (15% average reduction in heterozygotes *versus* 19% in homozygotes). The reductions of DISC1 gene expression levels, although modest, may, through the hub function of DISC1, exert subtle but pervasive, and thus neurodevelopmentally and physiologically relevant, effects.

Further, our analysis has added to the evidence for the DISC1 pathway having a role in the regulation of cytoskeletal function, synaptogenesis, and neurodevelopment [5] and sensory perception. This evidence pertains to normal variance in gene expression, but may also be clinically relevant and could be tested by association studies of target genes and expression studies of post-mortem brain tissue. Intriguingly, ‘cAMP mediated signaling’ appears as an overrepresented term for the NDE1 variant rs4781678 (Table S4) and not, as might have been expected, for a PDE4B or PDE4D variant. We have however recently demonstrated that DISC1, PDE4 and NDE1 co-associate and co-localize at the synapse, suggesting a role for NDE1 in co-modulating PDE4 dependent cAMP levels [29]. Sensory perception is a novel, emergent finding that fits well with the underlying concepts in psychosis of altered perception and salience [30]. This finding provides a new avenue for experimentation, for example in the analysis of mice mutant for Disc1 pathway genes [6]–[9].

The identification of seven psychoactive drug targets is noteworthy. To the best of our knowledge, these were identified and developed without any prior knowledge of a biological link to the DISC1 pathway *per se*. We argue from this that the DISC1 pathway offers a valid and circumscribed target for additional drug target development. Moreover and importantly, our analysis suggests that DISC1 pathway variant profiling may serve as a useful predictor of individual response to a given therapeutic.

The approach we have taken (Figure 1) can be adapted to any chosen gene or pathway. We suggest that this may prove a useful strategy for independent evaluation of candidate genes, their likely contribution to disease variance, their potential utility as therapeutic targets and as predictors of response to treatment.

## Materials and Methods

Multiple publicly available data sets were used to test the effects of genetic variants on genome-wide gene expression levels. Genotype and phased haplotype data were collected from the HapMap project ([31]; www.hapmap.org/), whilst expression data were collected from the NCBI Gene Expression Omnibus (GEO) database (www.ncbi.nlm.nih.gov/geo/ ) for studies that have used lymphoblastoid cell lines, where the individuals used partially or completely overlapped with the individuals included in the HapMap project.

### Expression data

The expression data came from three independent laboratories and can be obtained from the NCBI GEO database under the following identification codes: GDS2106 [17], GDS1048 [18], and GSE6536 [16]. One group had performed a replication analysis for their samples (GDS2106), which we used here as a fourth comparator being a technical replicate for the observations in GDS2106. Data were derived from different gene chip platforms: GDS2106 used Affymetrix GeneChip Human HG-Focus Target Array, GDS1048 used a Rosetta platform, and GSE6536 used the Illumina Sentrix Human-6 Expression BeadChip. The Affymetrix platform was re-annotated using a custom GeneChip library file (CDF file) [32]. Each study tested a different and partially overlapping sub-set of the CEU individuals (Figure S3).

### Statistical analysis

All variants considered to be ‘cis’ to the DISC1 gene (HapMap Build 35 co-ordinates, Chr1:227961133..228493750, 10 kb upstream of the immediately adjacent gene TSNAX to 10 kb downstream of DISC1) were tested for association to variance in the expression levels of DISC1 using the one-way ANOVA function in SPSS (version 14.0 for Windows). This was performed for all four HapMap populations using the expression data from the GEO dataset GSE6536. It was predicted that if a genomic area truly regulates DISC1 expression, then that region would be detected to associate in all populations. These findings were mapped back on to the linkage disequilibrium (LD) structure of this region. The pattern of LD was defined using the solid spine of LD (D′>0.8) to form haplotype blocks. Neighboring haplotype blocks with a Hedrick's multiallelic D′>0.9 [33] were joined to form one haplotype block.

Tests for association between genetic variants and gene expression were performed using the MA-ANOVA program in R, for each variant (n = 24) on each platform (n = 4, total of 96 genome wide analyses). The datasets ranged from 24 to 60 individuals analyzed. To take account of these multiple tests and between study variables, only those genes that displayed significant differences in expression (p<0.05) in all datasets on which it was measured and which changed in the same direction were considered robust and taken forward. This produced a list of genes that were differentially expressed for each DISC1 pathway variant. To determine whether the number of genes observed was greater than that expected by chance we calculated how many genes would be expected to show affected expression using a the 5% level for each platform. Since we were only looking for genes replicated across platforms we used an expected level of 0.25% (0.05×0.05) for genes on two platforms (10249 genes: 0.05×0.05×10249 = 25.62), an expected level of 0.0125% (0.05×0.05×0.05) for genes on three platforms (545 genes: 0.05×0.05×0.05×545 = 0.068) and an expected level of 0.000625% (0.05×0.05×0.05×0.05) for genes on four platforms (2351 genes: 0.05×0.05×0.05×0.05×2351 = 0.015). This resulted in an expected value of 25.71 genes identified per variant.

### Gene Ontology Analysis

The list of genes differentially expressed for each variant was uploaded into the web based Gene Ontology Tree Machine ([27]; http://bioinfo.vanderbilt.edu/gotm/ ) and the hypergeometric test utilized by GOTM to test for over representation of genes of similar function. GOTM tests were also applied to gene lists identified by more than one variant within the following categories, a) DISC1 expression altering variants, b) NDE1 variants, c) all variants that associate with major mental illness, d) all variants from the DISC1 pathway. The list of observed differentially expressed genes was compared to a reference gene list of all genes that had targets on multiple platforms, to determine if there was significant enrichment of gene ontology terms. The GOTM website is updated periodically and can not be searched using prior data assemblies. The analysis performed here was on the data assembly as it stood on 8th May 2008.

## Supporting Information

Figure S1 Illustration from the UCSC genome browser to show the locations of the DISC1 cis SNPs tested for association to DISC1 expression in relation to the LD structure in the four populations, the location of the DISC1 coding sequence and potential regulatory elements. Track 1, shows UCSC chromosomal location from the March 2006 build. Track 2 shows the chromosomal band. Track 3 shows the location of DISC1. Tracks 4, 6, 8 and 10 show the LD block structure in the four populations (alternating colors are for clarity). Tracks 5, 7, 9 and 11 show the SNPs used in the analysis and whether they associated below the 0.05 level of significance (Black SNPs are significant at the 0.05 level, Grey SNPs are not). Tracks 12 to 14 show the locations of potential regulatory elements in this region. a) Whole region analyzed. b) Close up of the 5′ end of DISC1 where consistent significant findings were observed.
(2.69 MB TIF)

Figure S2 Gene ontology tree illustrations, parts a) to w), from the Gene Ontology Tree Machine ([1]; http://bioinfo.vanderbilt.edu/gotm/ ) for the genes identified to be significantly differentially expressed by each variant and the four variant groupings used in this study. Ontological functions illustrated in red are significantly over represented in the genes whose expression levels are altered by that variant. a) DISC1 rs1765778 b) DISC1 rs1025526 c) DISC1 rs6541280 d) DISC1 rs3738398 e) DISC1 rs823167 f) DISC1 rs3738401 g) DISC1 rs821616 h) DISC1 interplay model i) NDE1 rs4781678 j) NDE1 rs2242549 k) NDE1 rs881803 l) NDE1 rs2075512 m) NDE1 haplotype n) NDEL1 haplotype o) PDE4B haplotype (Haplotype of rs4503327, rs2503222 and rs6588186) p) PDE4B rs7412571 q) PDE4B Haplotype (Haplotype of rs2503166, rs583018 and rs526772) r) PDE4B rs2503177 s) PDE4D rs1120303 t) DISC1 overlapping genes for DISC1 expression altering variants u) NDE1 overlapping genes v) DISC1 pathway associating variants overlapping genes w) DISC1 pathway all variants overlapping genes. 1.→Zhang B, Schmoyer D, Kirov S, Snoddy J (2004) GOTree Machine (GOTM): a web-based platform for interpreting sets of interesting genes using Gene Ontology hierarchies. BMC Bioinformatics 5: 16.
(10.22 MB TIF)

Figure S3 Venn diagram of the numbers of overlapping CEU individuals in the three publicly available expression datasets.
(1.48 MB TIF)

Table S1 Table to show the association between cis variants of DISC1 and DISC1 gene expression in the four HapMap populations. P-values in bold are below 0.05.
(0.12 MB XLS)

Table S2 Variants from the DISC1 pathway used in analysis of correlations with global gene expression levels.
(0.02 MB XLS)

Table S3 Table to show the number of genes significantly altered by each variant and the direction and fold change of effect for each gene.
(0.18 MB XLS)

Table S4 Table to show the gene ontology terms that are significantly enriched in the gene lists for each variant and the four groupings of variants.
(0.03 MB XLS)

Text S1 Unpublished article. Tomppo L, Hennah W, Lahermo P, Loukola A, Tuulio-Henriksson A, et al. (2009) Genes of the DISC1 Pathways in the Etiology of Schizophrenia. Biol Psychiatry: (in press)
(0.81 MB PDF)
