# Supplementary figures and images for: The DISC1 Pathway Modulates Expression of Neurodevelopmental, Synaptogenic and Sensory Perception Genes
Source: PLoS One. 2009 Mar 20;4(3):e4906. doi: 10.1371/journal.pone.0004906 (PMC2654149; doi:10.1371/journal.pone.0004906)

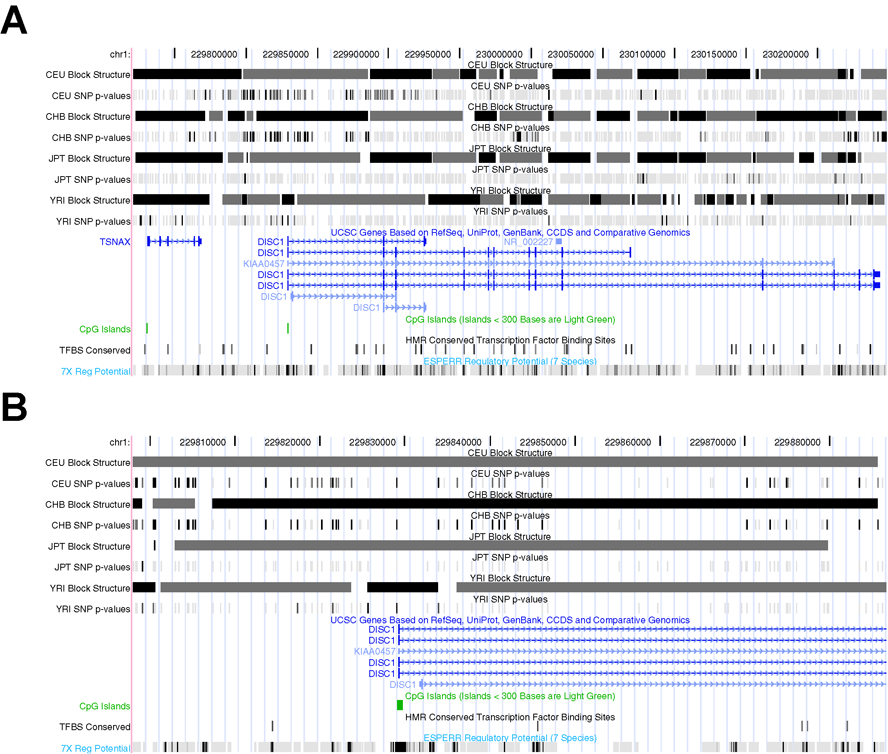

Supplement: Figure S1 — Illustration from the UCSC genome browser to show the locations of the DISC1 cis SNPs tested for association to DISC1 expression in relation to the LD structure in the four populations, the location of the DISC1 coding sequence and potential regulatory elements. Track 1, shows UCSC chromosomal location from the March 2006 build. Track 2 shows the chromosomal band. Track 3 shows the location of DISC1. Tracks 4, 6, 8 and 10 show the LD block structure in the four populations (alternating colors are for clarity). Tracks 5, 7, 9 and 11 show the SNPs used in the analysis and whether they associated below the 0.05 level of significance (Black SNPs are significant at the 0.05 level, Grey SNPs are not). Tracks 12 to 14 show the locations of potential regulatory elements in this region. a) Whole region analyzed. b) Close up of the 5′ end of DISC1 where consistent significant findings were observed. (2.69 MB TIF) [file pone.0004906.s001.tif]

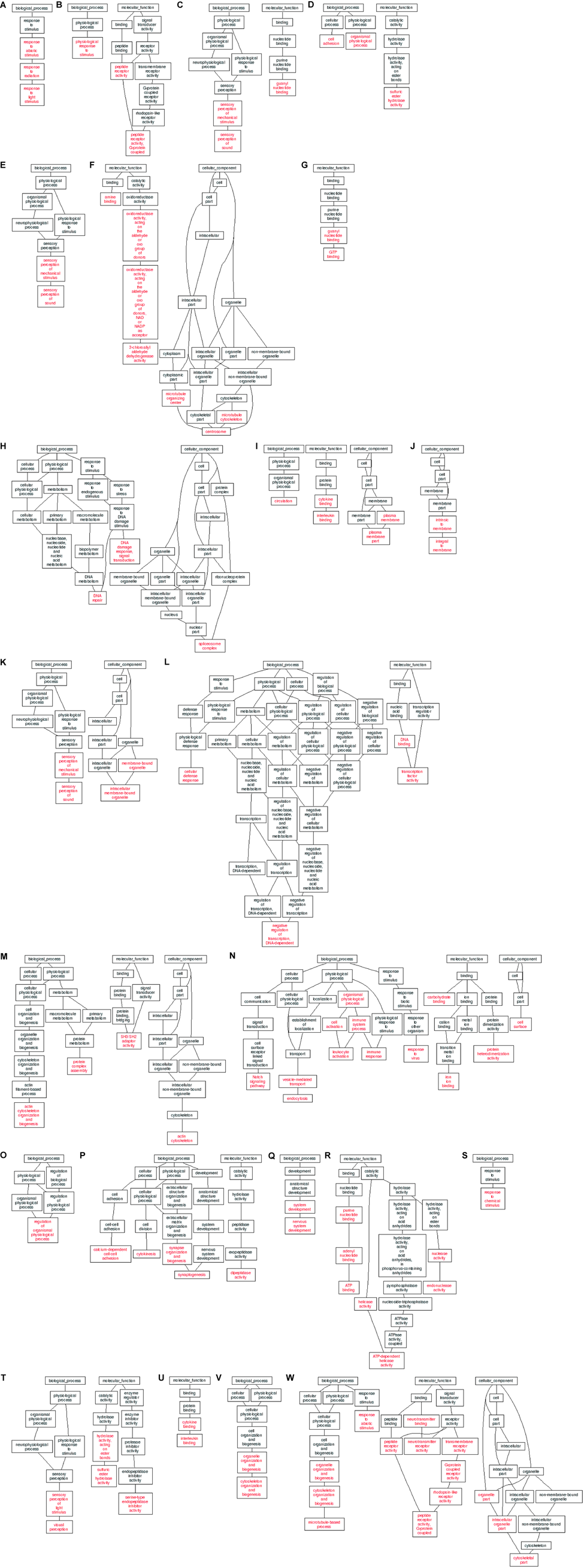

Supplement: Figure S2 — Gene ontology tree illustrations, parts a) to w), from the Gene Ontology Tree Machine ([1]; http://bioinfo.vanderbilt.edu/gotm/ ) for the genes identified to be significantly differentially expressed by each variant and the four variant groupings used in this study. Ontological functions illustrated in red are significantly over represented in the genes whose expression levels are altered by that variant. a) DISC1 rs1765778 b) DISC1 rs1025526 c) DISC1 rs6541280 d) DISC1 rs3738398 e) DISC1 rs823167 f) DISC1 rs3738401 g) DISC1 rs821616 h) DISC1 interplay model i) NDE1 rs4781678 j) NDE1 rs2242549 k) NDE1 rs881803 l) NDE1 rs2075512 m) NDE1 haplotype n) NDEL1 haplotype o) PDE4B haplotype (Haplotype of rs4503327, rs2503222 and rs6588186) p) PDE4B rs7412571 q) PDE4B Haplotype (Haplotype of rs2503166, rs583018 and rs526772) r) PDE4B rs2503177 s) PDE4D rs1120303 t) DISC1 overlapping genes for DISC1 expression altering variants u) NDE1 overlapping genes v) DISC1 pathway associating variants overlapping genes w) DISC1 pathway all variants overlapping genes. 1.→Zhang B, Schmoyer D, Kirov S, Snoddy J (2004) GOTree Machine (GOTM): a web-based platform for interpreting sets of interesting genes using Gene Ontology hierarchies. BMC Bioinformatics 5: 16. (10.22 MB TIF) [file pone.0004906.s002.tif]

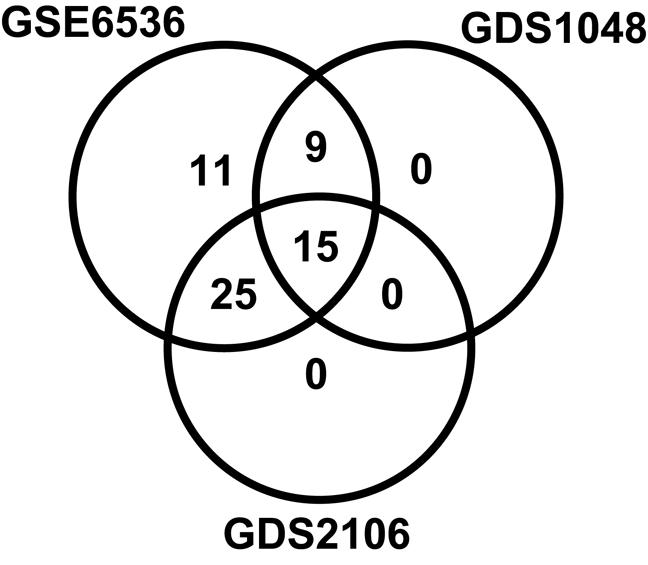

Supplement: Figure S3 — Venn diagram of the numbers of overlapping CEU individuals in the three publicly available expression datasets. (1.48 MB TIF) [file pone.0004906.s003.tif]
