# Supplementary material for: The DISC1 Pathway Modulates Expression of Neurodevelopmental, Synaptogenic and Sensory Perception Genes
Source: PLoS One. 2009 Mar 20;4(3):e4906. doi: 10.1371/journal.pone.0004906 (PMC2654149; doi:10.1371/journal.pone.0004906)
Supplement: Text S1 — Unpublished article. Tomppo L, Hennah W, Lahermo P, Loukola A, Tuulio-Henriksson A, et al. (2009) Genes of the DISC1 Pathways in the Etiology of Schizophrenia. Biol Psychiatry: (in press) (0.81 MB PDF) [file pone.0004906.s008.pdf]

Elsevier Editorial System(tm) for Biological Psychiatry
Manuscript Draft

Manuscript Number: BPS-D-08-01306R1

Title: Association between genes of DISC1 interactors and schizophrenia supports the role of the DISC1 pathway in the etiology of major mental illnesses

Article Type: Archival Report

Keywords: schizophrenia; DISC1; NDEL1; PDE4D; endophenotype

Corresponding Author: Professor Leena Peltonen, MD, PhD

Corresponding Author's Institution: National Public Health Institute

First Author: Liisa Tomppo

Order of Authors: Liisa Tomppo; William Hennah, PhD; Päivi Lahermo, PhD; Anu Loukola, PhD; Annamari Tuulio-Henriksson, PhD; Jaana Suvisaari, MD, PhD; Timo Partonen, MD, PhD; Jesper Ekelund, MD, PhD; Jouko Lönnqvist, MD, PhD; Leena Peltonen, MD, PhD

Abstract: BACKGROUND: Disrupted in Schizophrenia 1 (DISC1) is currently one of the most interesting candidate genes for major mental illness, having been demonstrated to associate with schizophrenia, bipolar disorder, major depression, autism, and Asperger's syndrome. We have previously reported a DISC1 haplotype, HEP3, and an NDE1 spanning tag haplotype to associate to schizophrenia in Finnish schizophrenia families. Since both DISC1 and NDE1 display association in our study sample, we hypothesized that other genes interacting with DISC1 may also have a role in the etiology of schizophrenia.
METHODS: We selected 11 additional genes encoding components of the "DISC1 pathway" and studied these in our study sample of 476 families including 1857 genotyped individuals. We performed SNP and haplotype association analyses in two independent sets of families. For markers and haplotypes found to be consistently associated in both sets, the overall significance was tested using the combined set of families.

RESULTS: We identified three SNPs to be associated with schizophrenia in PDE4D (rs1120303, p = 0.021), PDE4B (rs7412571, p = 0.018) and NDEL1 (rs17806986, p = 0.0038). Greater significance was observed with allelic haplotypes of PDE4D (p = 0.00084), PDE4B (p = 0.0022 and p = 0.029) and NDEL1 (p = 0.0027) that increased or decreased schizophrenia susceptibility.
CONCLUSIONS: Our findings with other converging lines of evidence support the underlying importance of DISC1-related molecular pathways in the etiology of schizophrenia and other major mental illnesses.

Response to Reviewers: Response to reviewers' comments

Reviewer 1

The reviewer asks for clarification as to whether one or more affected individuals from each family were included in the analyses. As described in the results section, initially all the affected individuals were included. If a marker or a haplotype displayed significant evidence for association when tested in stage 1 and stage 2 samples the overall significance was tested using both all affected individuals and one affected individual per family (page 13, paragraph 1). Since association was observed outside of the family structure and the confounding factor of linkage, segregation of these variants has not been pursued.

The reviewer also asks for clarification within the figure legends. There we state that SNPs with rs-numbers indicate SNPs that display association to schizophrenia, either independently or in haplotypes. All the haplotype blocks (according to LD structure) were tested for association (as indicated in the text, page 9, paragraph 3). This is now stated more clearly in the figure legend.

The reviewer's minor comments have been all been included into the manuscript.

Reviewer 3

The reviewer asks if our study design needs to take into account multiple test correction by Bonferroni correction. Since we elected for a two-stage study design that allows for replication of observations in an independent study sample multiple test correction is not required.

The reviewer wonders also if we detected any association between bipolar disorder and the genes analyzed. Our sample includes also individuals who are affected with bipolar disorder and are siblings to individuals with schizophrenia. However, these individuals were not included in our family based analyses since it has been shown that in families ascertained for schizophrenia the genetic liability for disorders in both schizoaffective disorder and schizophrenia spectrum disorders is increased, whereas bipolar disorder, despite having some overlap in genetic risk, display the same liability as the general population background. Thus no analyses were performed using these individuals. The number of individuals with bipolar disorder in our sample is too small (N=67) to perform separate association analysis for this disorder. We therefore hope that through publication of this work, independent researchers will study these genes with other mental illnesses, including bipolar disorder.

The additional references suggested by the reviewer have been added to the manuscript.

Reviewer 5

The reviewer comments on our use of the term "pathway" in the hypothesis of the "DISC1 pathway", suggesting that "network" is more representative of the genes we have studied. The "DISC1 pathway" hypothesis proposes that genes related to DISC1 may also be disrupted in major mental illnesses, by producing disruptions to the same pathways as risk variants in DISC1. As the majority of the functions and roles of DISC1 through its "hub" effects are yet to be formalized, we do not reliably know what genes are of interest in relevant pathways. We have for this study therefore concentrated our focus on the direct interactors of DISC1, and fully admit that this is more of network than a pathway. However, since we use the term "DISC1 pathway" to refer to the global hypothesis rather than just to these selected genes we feel that our use of the term is justified.

The reviewer requires further discussion on type I and type II errors. The statistical methodology we have used is widely used in genetic studies and has been stated to effectively increase true positives (Wen et al: A two stage design for multiple testing in large-scale association studies, J Hum Genet, 2006 and van den Oord et al: A framework for controlling false discovery rates and minimizing the amount of genotyping in search for disease mutations, Trends Genet, 2003). Although the use of a smaller sample in Stage 1 may be thought to increase the potential for false negatives, we feel this is balanced by the increase in true positives. Further, since the use of traditional multiple test correction is considered to be overly conservative, it can be argued that a

two stage design actually decreases false negatives (Wen et al). This is clarified in the manuscript (page 9, paragraph 1).

Kansanterveyslaitos
Folkhälsoinstitutet
National Public Health Institute

Biological Psychiatry
John H. Krystal
Yale University School of Medicine and VA Connecticut Healthcare System
West Haven, CT
USA

Re: BPS Submission: BPS-D-08-01306

Dear Professor Krystal,

Thank you for the favorable comments regarding our manuscript "Association between genes of DISC1 interactors and schizophrenia supports the role of the DISC1 pathway in the etiology of major mental illnesses" that we submitted for publication in your esteemed journal.

We have now revised the manuscript according to the constructive and insightful suggestions by the reviewers. We feel that we have addressed all the concerns raised by the reviewers and would kindly ask you to consider this revised manuscript for publication. The responses to the reviewers' comments are provided below.

Yours sincerely,

Leena Peltonen, MD, PhD
Head of Human Genetics
Wellcome Trust Sanger Institute
Hinxton Cambridge UK
Department of Medical Genetics, University of Helsinki
Department of Molecular Medicine, National Public Health Institute
Biomedicum Helsinki, Finland
The Broad Institute, MIT & Harvard University
Cambridge, MA,USA

tel: +358-9-4744-8393
fax: +358-9-4744-8480

Response to reviewers' comments

Reviewer 1

The reviewer asks for clarification as to whether one or more affected individuals from each family were included in the analyses. As described in the results section, initially all the affected individuals were included. If a marker or a haplotype displayed significant evidence for association when tested in stage 1 and stage 2 samples the overall significance was tested using both all affected individuals and one affected individual per family (page 13, paragraph 1). Since association was observed outside of the family structure and the confounding factor of linkage, segregation of these variants has not been pursued.

The reviewer also asks for clarification within the figure legends. There we state that SNPs with rs-numbers indicate SNPs that display association to schizophrenia, either independently or in haplotypes. All the haplotype blocks (according to LD structure) were tested for association (as indicated in the text, page 9, paragraph 3). This is now stated more clearly in the figure legend.

The reviewer's minor comments have been all been included into the manuscript.

Reviewer 3

The reviewer asks if our study design needs to take into account multiple test correction by Bonferroni correction. Since we elected for a two-stage study design that allows for replication of observations in an independent study sample multiple test correction is not required.

The reviewer wonders also if we detected any association between bipolar disorder and the genes analyzed. Our sample includes also individuals who are affected with bipolar disorder and are siblings to individuals with schizophrenia. However, these individuals were not included in our family based analyses since it has been shown that in families ascertained for schizophrenia the genetic liability for disorders in both schizoaffective disorder and schizophrenia spectrum disorders is increased, whereas bipolar disorder, despite having some overlap in genetic risk, display the same liability as the general population background. Thus no analyses were performed using these individuals. The number of individuals with bipolar disorder in our sample is too small (N=67) to perform separate association analysis for this disorder. We therefore hope that through publication of this work, independent researchers will study these genes with other mental illnesses, including bipolar disorder.

The additional references suggested by the reviewer have been added to the manuscript.

Reviewer 5

The reviewer comments on our use of the term "pathway" in the hypothesis of the "DISC1 pathway", suggesting that "network" is more representative of the genes we have studied. The "DISC1 pathway" hypothesis proposes that genes related to DISC1 may also be disrupted in major mental illnesses, by producing disruptions to the same pathways as risk variants in DISC1. As the majority of the functions and roles of DISC1 through its "hub" effects are yet to be formalized, we do not reliably know what genes are of interest in relevant pathways. We have for this study therefore concentrated our focus on the direct interactors of DISC1, and fully admit that this is

more of network than a pathway. However, since we use the term “DISC1 pathway” to refer to the global hypothesis rather than just to these selected genes we feel that our use of the term is justified.

The reviewer requires further discussion on type I and type II errors. The statistical methodology we have used is widely used in genetic studies and has been stated to effectively increase true positives (Wen et al: A two stage design for multiple testing in large-scale association studies, J Hum Genet, 2006 and van den Oord et al: A framework for controlling false discovery rates and minimizing the amount of genotyping in search for disease mutations, Trends Genet, 2003). Although the use of a smaller sample in Stage 1 may be thought to increase the potential for false negatives, we feel this is balanced by the increase in true positives. Further, since the use of traditional multiple test correction is considered to be overly conservative, it can be argued that a two stage design actually decreases false negatives (Wen et al). This is clarified in the manuscript (page 9, paragraph 1).

Association between genes of DISC1 interactors and schizophrenia supports the role of the DISC1 pathway in the etiology of major mental illnesses

Liisa Tomppo (1), William Hennah, PhD (1,2), Päivi Lahermo, PhD (3), Anu Loukola PhD (1,3), Annamari Tuulio-Henriksson PhD (4,5), Jaana Suvisaari, MD, PhD (4), Timo Partonen, MD, PhD, (4), Jesper Ekelund, MD, PhD (1,4,6), Jouko Lönnqvist, MD, PhD (4,6), Leena Peltonen, MD, PhD (1,7,8,9)

1. FIMM Institute for Molecular Medicine and National Public Health Institute, Biomedicum, P.O.Box 104, FIN-00251, Helsinki, Finland
2. Medical Genetics Section, University of Edinburgh, EH4 2XU, Edinburgh, UK
3. Finnish Genome Center, University of Helsinki, Helsinki, Finland
4. Department of Mental Health and Alcohol Research, National Public Health Institute, 00300, Helsinki, Finland
5. Department of Psychology, University of Helsinki, Finland
6. Department of Psychiatry, Helsinki University Central Hospital, 00029, Helsinki, Finland
7. Department of Medical Genetics, University of Helsinki, Helsinki, Finland
8. The Broad Institute, MIT, Boston, USA
9. Wellcome Trust Sanger Institute, Genome Campus, Hinxton, CB10 1SA, UK

Corresponding author: Leena Peltonen, Department of Molecular Medicine, National Public Health Institute, PO Box 104, 00251, Helsinki, Finland. Tel: +358 9 4744 8393; Fax: +358 9 4744 8480; Email: leena.peltonen@ktl.fi

Key words: schizophrenia, DISC1, NDEL1, PDE4D, PDE4B, endophenotype

Word count abstract: 232

Word count text: 3855

ABSTRACT

BACKGROUND: Disrupted in Schizophrenia 1 (DISC1) is currently one of the most interesting candidate genes for major mental illness, having been demonstrated to associate with schizophrenia, bipolar disorder, major depression, autism, and Asperger's syndrome. We have previously reported a DISC1 haplotype, HEP3, and an NDE1 spanning tag haplotype to associate to schizophrenia in Finnish schizophrenia families. Since both DISC1 and NDE1 display association in our study sample, we hypothesized that other genes interacting with DISC1 may also have a role in the etiology of schizophrenia.

METHODS: We selected 11 additional genes encoding components of the "DISC1 pathway" and studied these in our study sample of 476 families including 1857 genotyped individuals. We performed SNP and haplotype association analyses in two independent sets of families. For markers and haplotypes found to be consistently associated in both sets, the overall significance was tested using the combined set of families.

RESULTS: We identified three SNPs to be associated with schizophrenia in PDE4D (rs1120303, $p = 0.021$), PDE4B (rs7412571, $p = 0.018$) and NDEL1 (rs17806986, $p = 0.0038$). Greater significance was observed with allelic haplotypes of PDE4D ($p = 0.00084$), PDE4B ($p = 0.0022$ and $p = 0.029$) and NDEL1 ($p = 0.0027$) that increased or decreased schizophrenia susceptibility.

CONCLUSIONS: Our findings with other converging lines of evidence support the underlying importance of DISC1-related molecular pathways in the etiology of schizophrenia and other major mental illnesses.

INTRODUCTION

Disrupted in Schizophrenia 1 (DISC1) is one of the most promising candidate genes for schizophrenia. It was identified as disrupted by a balanced translocation (t(1;11)(q42.1;q14.3)) in a large Scottish family with high prevalence of schizophrenia and other psychiatric disorders (1, 2). Independent evidence for DISC1 was found by our work using Finnish cohorts. Initially we found strong and replicated linkage to 1q42 with markers intragenic of DISC1 (3, 4). We have since reported an association between allelic haplotypes of DISC1 and schizophrenia (5, 6), bipolar disorder (7), autism and Asperger's syndrome (8). Further studies of the most robust of these haplotypes identified in the original schizophrenia study, have implicated that it predisposes to schizophrenia especially in males (6) and associates with poorer visual working memory performance and reduced grey matter volume in the prefrontal cortex (9, 10). To date a number of groups have reported association findings between DISC1 and several neuropsychiatric disorders (7, 9, 11-20), neurocognitive (21-23) and neuroimaging (9, 24) phenotypes. DISC1 has also been strongly linked to neuronal development based on numerous indirect studies (9, 25-27).

DISC1 is considered a multifunctional "hub" for many protein interactions acting along several pathways (28). Proteins have been recognized to bind to DISC1 through yeast two-hybrid screens or co-immunoprecipitation experiments (26, 29-32). A number of these have since been identified as promising candidates for roles in the etiology of schizophrenia and other mental illnesses. We have previously reported an association between NDE1 and schizophrenia in Finnish schizophrenia families (33). Further, NDE1 and NDEL1 have displayed significant interplay with DISC1 that associates with the disorder (34). PDE4B was initially recognized as a candidate for schizophrenia at a translocation breakpoint between

chromosomes 1 and 16 in individuals with schizophrenia (35). Since, several groups have reported association findings between PDE4B and schizophrenia (36-38).FEZ1 has been reported to associate with schizophrenia in the Japanese population (39). This evidence has led to the concept of the “DISC1 pathway”, a hypothesis which proposes that disruption of the pathways DISC1 is involved in provides risk to mental illness, not just disruption of DISC1 itself (29, 39, 40).

In the present study, we aimed to investigate DISC1 binding partners as potential schizophrenia susceptibility genes in 476 Finnish families ascertained for schizophrenia. We included 11 genes coding for proteins convincingly reported to bind to and interact with DISC1 (18, 40) . These included NDEL1, PDE4B and FEZ1 mentioned above. Additionally we included PDE4D, PCNT, MAP1A, PAFAH1B1, TUBA1A, TRAF3IP1, ATF4 and ATF5.

Figure 1

MATERIAL & METHODS

Sample

In this study we used the same Finnish schizophrenia family sample that has previously been used in DISC1 related linkage and association analyses in Finland (33), plus an additional set of 18 families. The sample collection method has remained unchanged over the years. The individuals with schizophrenia (probands) are identified from three nationwide data sources; the hospital discharge, disability pension, and reimbursed medication registers (41). The first-degree family members of each proband were thereafter identified through the Population Register Centre, enabling the construction of pedigrees. Personal data recorded in the

Population information system is maintained by the Population Register Centre and local register offices include personal identity code, family relations and date of birth and death (if applicable). Altogether 33,371 individuals born between years 1940 and 1976, and diagnosed with schizophrenia (according to the ICD-8, DSM-III-R or ICD-10 classifications) between years 1969 and 1998, have been identified from these registers (33). Family information has been collected from the Finnish population register and pedigrees have been constructed. This study now includes 476 nuclear families that include a total 2756 individuals out of which 1857 have been genotyped in this study. The lifetime diagnosis for each case in the study sample has been evaluated according to Diagnostic and Statistical Manual of Mental Disorders forth edition (DSM-IV) criteria independently by two psychiatrists (42). Concordance between these two psychiatrists is high (kappa values range from 95% to 99% depending on liability class). However in the case of a disagreement, a third psychiatrist estimated life-time diagnosis and the consensus best-estimate life-time diagnosis was made. In addition to the proband with schizophrenia, family members with other psychiatric illnesses were also identified. It is therefore possible to define affection using increasingly inclusive liability classes (LC). LC1 consists of individuals diagnosed with schizophrenia, LC2 adds individuals diagnosed with schizoaffective disorder to the sample and LC3 adds individuals affected with schizophrenia spectrum disorders (33) (schizoid, schizotypal and paranoid personality disorders, schizophreniform, delusional and brief psychotic disorder, and psychosis not otherwise specified) , and LC4 adds individuals with bipolar affective disorder or major depression with and without the presence of psychosis. In this study we have restricted our end-state diagnosis phenotype to LC3. It has been shown that in families ascertained for schizophrenia the genetic liability for disorders in both LC2 and LC3 is increased (43), whereas the disorders in LC4, despite having some overlap in genetic risk, display the same liability as the general population background (43-45) . According to this

LC3 criterion our study includes a total of 886 affected individuals out of which 725 are genotyped.

In this present study, the family sample was randomly split into two non-overlapping sub-samples to be analyzed as exploratory and replication data sets. The exploratory set contained 171 families, and the replication set 305 families. The combined sample set included both sample sets. In addition, a sample of 57 anonymous Finnish parent-offspring trios, representing a random sample of the Finnish population was used as a control sample.

Neuropsychological test methods

For a sub-sample of 186 families a neuropsychological test battery has been administered. The tests were administered in a fixed order by experienced psychologists or especially trained psychiatric nurses, and the scoring of the tests was performed by experienced psychologists (46). The following variables were used in the analyses. (1) From the Wechsler Memory Scale –Revised (WMS-R) (47) we included Verbal and Visual Span forward subtests as measures of auditory and visual attention, respectively. The respective backward condition of these WMS-R subtests was used as measures of verbal and visual working memory. (2) From the California Verbal Learning Test (CVLT) (48), total recall from trials 1-5 were used as a measure of learning. The other included CVLT variables were semantic clustering as a measure of learning strategy, and short delay and long delay recall. (3) From the Wechsler Adult Intelligence Scale - Revised (WAIS-R) (49), the Vocabulary subtest was included as an estimate of basic ability, and the Digit Symbol subtest as a measure of information processing speed. The selected traits are either direct measures of learning and memory such as short delay memory, long delay memory, and verbal learning, or highly relevant to learning process such as auditory attention, visual working memory, verbal

working memory, verbal attention and semantic clustering. The test scores were normally distributed in our sample.

Genotyping methods

The SNPs were selected from the international HapMap project database, build #16, phase 1 (50). The LD structure for the 11 candidate genes was defined using all the SNPs with a minor allele frequency > 5% in the population of European descent (CEU, Utah residents with ancestry from Northern and Western Europe). We used the Haploview program (51) and the solid spine of LD criterion (D' > 0.8). LD blocks with a Hedricks multiallelic (52)D' ≥ 0.9 were combined. Haplotype tagging SNPs were prioritized to obtain the optimal coverage of the genes (number of SNPs selected = 91). Additional SNPs from the HapMap and Perlegen (53) databases were selected to provide coverage if genotypes of tagging SNPs were not of high enough quality (number of SNPs selected = 57; Total number of SNPs = 148). Genotyping was performed using the Sequenom platform according to manufacturer's recommendations (54). SNPs were later rejected if they had a genotyping success rate < 80% (6 SNPs). None of the genotyped SNPs showed deviation for Hardy-Weinberg disequilibrium (HWE) ($p > 0.001$). After these quality control measures 142 SNPs were included in the analyses (Supplementary Information Table 3).

Statistical methods

The SNP genotypes were checked for Mendelian errors using Pedcheck (55). In case of an error in any genotype, all the genotypes for that marker in the relevant family were discarded (Mendelian error rate: 0.000076 / genotype).

Association tests between affection status and gene variants were performed using a two-stage study design described above. Such a design has been demonstrated to increase the identification of true positives while also decreasing the rate of false negative associations compared to using the more traditional, but overly conservative, Bonferroni correction (56, 57).

Two-point analyses were performed using the program Pseudomarker (58). This program performs linkage as well as linkage disequilibrium analyses on samples of mixed form (families and trios being combined here), correcting for the effect of linkage on the association tests, and is able to deal with cases where parental genotypes are not known (58).

To perform haplotype analyses, we identified haplotype blocks according to the LD structure defined by Haploview, as described above. For NDEL1, TRAF3IP1, ATF4, ATF5, MAP1A, TUBA1A, FEZ1 and PCNT only one haplotype block for each gene was present. PAFAH1B1, PDE4B and PDE4D included 2, 10 and 17 LD blocks, respectively. In ATF4, PDE4B and PDE4D we were able to tag 0, 8 and 16 blocks respectively, in other genes, all the blocks were tagged. Generally, the LD structure in our sample correlates well with the LD structure defined using the HapMap CEU population. However, for NDEL1 the LD pattern in the CEU population predicts two LD blocks meanwhile according to our sample one block was predicted. Hedricks D' between the blocks defined according to CEU population in our sample is 1.00 (compared to 0.39 in CEU population). Therefore we analyzed these blocks as one.

Haplotype association analyses to end-state diagnosis were performed using the two-stage method described previously. We compared the frequencies of the haplotypes in the affected

offspring of the schizophrenia families to that of the founders of the control trios. These frequencies were defined by estimating each individual's most likely haplotypes using Simwalk2, a Markov chain Monte Carlo (MCMC) and simulated annealing program (59). Only haplotype block tagging SNPs were taken into account when constructing the haplotypes. Haplotype association analyses to end-state diagnosis were performed using the chi squared test, testing each possible haplotype against all other haplotypes combined in a 2 x 2 table. A global test was performed in a 2 x n (n = number of alleles) table for the haplotype blocks including one or more haplotypes displaying significant association to affection status in both sample sets. Haplotypes with frequencies < 5% in both the case and control samples where combined together to avoid the deviation that these rare haplotypes might have on the result

The SNPs and haplotypes showing evidence for association to end state diagnosis ($p < 0.05$) in our two stage design were tested for the association with quantitative neurocognitive traits (622 individuals available with quantitative data). These analyses were performed using the QTDT program and the orthogonal model (60, 61). Here the haplotypes were re-coded to form a "bi-allelic" marker, so as to test the hypothesized variant against all others combined. We used age, sex and affection status according to LC3 as covariates in our analysis.

The study has been accepted by the Ministry of Social Affairs and Health (Finland) and institutional review boards. All subjects have provided written informed consent.

RESULTS

We analyzed single SNP markers and haplotypes for their association to schizophrenia using a two stage study design with random sampling of our original 476 Finnish families. Association analyses between affection status and SNPs and haplotypes were first performed in a sample of 171 families. SNPs and haplotypes displaying evidence for association with p-values < 0.05 proceeded to analyses in a second sample consisting of 305 independent families. Markers and haplotypes passing the replication criteria in the second stage were analyzed using both sample sets combined.

In stage one 14 SNPs and 19 haplotypes in NDEL1, PDE4B, PDE4D, PAFAH1B1, MAP1A and TRAF3IP1 met our criteria for progressing to stage two. No markers or haplotypes in FEZ1, PCNT, TUBA1A, ATF4 and ATF5 met this criterion. In stage two associations with three markers and four haplotypes of NDEL1, PDE4D and PDE4B were replicated, the same allele showing association with the disorder in both sample sets. These markers and haplotypes were then tested for their overall significance in the combined sample set (Table 1, Supplementary Information Table 1 and Supplementary Information Table 2).

Table 1

Those SNPs and haplotypes that passed through the two stages are as follows. In PDE4D, rs1120303 showed association with an overall p-value of 0.021 (the minor allele frequency in schizophrenia family founders, $MAF_{scz} = 0.13$ versus the minor allele frequency in control family founders, $MAF_{control} = 0.19$; Block 15, 65 kb; Minor allele = T) in the combined dataset. The haplotype block in which this SNP is located also provided evidence of association with a haplotype comprising the GGACA alleles of SNPs rs13190249, rs1120303, rs921942, rs10805515 and rs10514862 being significantly over-represented in affected

individuals ($p = 0.00084$, frequency in affected individuals, $F_{affected} = 0.40$, frequency in controls, $F_{control} = 0.28$, global p-value 0.0034). The SNP rs7412571 in PDE4B provided overall association with a p-value of 0.018 ($MAF_{scz} = 0.45$, $MAF_{control} = 0.35$; Block 5, 76 kb; Minor allele = T). Although the haplotype block in which the rs7412571 SNP is located did not add any further evidence of association its neighboring block does provide such evidence. Block 4 (104 kb) comprises the SNPs rs4503327, rs2503222 and rs6588186, with two alleles displaying evidence for association in two sets of families and in the combined dataset. The haplotype consisting of the CCC allele was over-represented in affected offspring with an overall p-value of 0.029 ($F_{affected} = 0.28$, $F_{control} = 0.21$) while the CTT haplotype of this block was under-represented with overall p-value of 0.0022 ($F_{affected} = 0.022$, $F_{control} = 0.058$). The overall global p-value for this block in the combined sample was 0.0060. In NDEL1, the rs17806986 SNP, located close to the 5' end of the gene showed association with an overall p-value of 0.0038 ($MAF_{scz} = 0.27$, $MAF_{control} = 0.37$; Minor allele = C). The CGCG haplotype of SNPs rs17806986, rs1391768, rs1391766 and rs3817003 extending over the whole gene was under-represented in affected individuals ($p = 0.0027$, $F_{affected} = 0.12$, $F_{control} = 0.19$, global $p = 0.033$). (Figure 2)

Figure 2

Previous reports of DISC1 (6) and the DISC1 pathway genes (33, 38) have displayed distinct sex differences in their allelic association. We tested for any potential sex differences in allele frequencies of the recognized associating variants in our sample and could detect no differentiating affects between female and male offspring in these Finnish families (Table 2).

Table 2

Since some of the affected individuals in the sample are siblings, we also tested the identified haplotype variants using only one randomly selected affected offspring per family to account for the possible confounding effect of linkage in the obtained results. Three out of the four haplotypes remained significantly associated with the following p-values: PDE4D = 0.0055, NDEL1 = 0.0018 and PDE4B protective and risk = 0.0025 and 0.070 respectively.

Since DISC1 has been previously shown to be associated with visual working memory in these families (10), and with other quantitative neurocognitive traits in other sample sets, we wanted to test these newly identified variants for their association with such traits. In addition to visual working memory, we included several learning and memory related variables in the analyses. Due to the reduction of the neuropsychologically tested sample size we tested all the recognized SNPs and haplotypes only in the combined sample. Of the seven variants, none associated significantly ($p < 0.05$) with any of the nine tested traits.

DISCUSSION

We show here that three genes involved in the same intracellular pathways with DISC1 associate significantly with schizophrenia in a Finnish family sample. We identified SNP and haplotype variants in PDE4B, PDE4D and NDEL1 that were either under-represented or over-represented in families ascertained for schizophrenia. The minor allele of rs7412571 in PDE4B was significantly over-represented in affected individuals. The neighboring haplotype block displayed both an over-represented ("risk") and an under-represented ("protective") allele. However the over-represented haplotype did not remain significantly associated when we tested only one affected offspring per family. This would suggest that the under-

represented haplotype is of greater importance. PDE4D displayed significant association to schizophrenia with a SNP whose minor allele was significantly under-represented in individuals with schizophrenia. Consistent with this finding, a haplotype including the major allele of this same SNP was significantly over-represented in the cases. The minor allele at the NDEL1 rs17806986 SNP displayed significant under representation to schizophrenia. This SNP was a part of the NDEL1 gene spanning haplotype that was also significantly under-represented in affected individuals.

Fatemi and colleagues have recently reported on an association between schizophrenia and the major allele of SNP rs1354064 in PDE4B that is located in the same LD block we detected association with (36). We had not included this SNP in this study but our SNP rs2503222 tags the variation of this SNP ($r^2 = 0.82$). Even though we did not see evidence for this SNP alone, our haplotype finding further supports the importance of this locus. Further, a recent report by Pickard and colleagues also noted a haplotype within PDE4B in the Scottish population to be protective against schizophrenia (38). Although this haplotype is located approximately 70 kb toward the 3' end compared to our haplotype finding, and in an independent LD block, we were able to detect association in the same region as the Scottish haplotype, with the SNP rs7412571, suggesting that the same region might also be involved in the etiology of schizophrenia in the Finnish population. The minor allele of this SNP was over-represented in patients with schizophrenia. This SNP tags ($r^2 = 1$) two SNPs included in the Scottish haplotype. Consistent with our finding, the major allele of these two SNPs were included in the protective haplotypes in the Scottish study. Two previous reports have highlighted protective SNPs recognized close to 3' end of the gene, although we did not detect any evidence for association in this region (36, 37).

Burdick et al have reported on an association with NDEL1 to schizophrenia. They recognized an NDEL1 spanning haplotype being under-represented in affected individuals and further reported that the G allele of SNP rs1391768 was over represented in affected individuals (34). In contrast, in our sample this G allele is part of a NDEL1 spanning putative protective haplotype.

NDEL1 has been a strong schizophrenia susceptibility candidate gene (27, 62) based on its role in neuronal migration and neuronal outgrowth (63) . Further, it is part of the same Dynein signaling pathway as PAFAH1B1, another DISC1 binding partner (64), and RELN (65), a protein that is also implicated in the etiology of schizophrenia (66). Previously NDEL1 and its homologue NDE1 have been strongly linked to prenatal and early age neuronal development (63, 64, 67) but recent findings suggest a wider role for NDEL1(68). According to recent findings, DISC1 has a central role in adult neurogenesis along with NDEL1 in mice. Our finding proposes the involvement of these genes also in the etiology of adult onset disorders. Further, involvement of these genes in the etiology of schizophrenia would support the hypothesis that the vulnerability for developing these disorders might originate already during embryonic development.

We are the first to report direct association between PDE4D and schizophrenia. Interestingly PDE4D (5q11.2-5q12.1) was located close to a linkage peak (chromosome 5q12.3, LOD = 2.59) in our previous genome wide linkage scan when the analysis was conditioned by absence of previously recognized DISC1 risk haplotype HEP3 (33).

Our findings thus support the previous reports especially for PDE4B. Even though our findings are encouraging, naturally further studies are needed to establish the relevance of these genes in the etiology of psychiatric disorders.

It is well established that patients with schizophrenia have several specific memory deficits (69). Given the evidence supporting the involvement of PDE4B and PDE4D in the etiology of schizophrenia (35-38) and their supposed involvement in memory functions (70, 71), it was of interest to investigate if PDE4D and PDE4B would, in addition to association with schizophrenia, display association with learning and memory related quantitative traits. However, no significant association was detected with the recognized variants. Yet this may be due to the reduced sample size in the quantitative trait analyses compared to association tests using affection status.

Since all these genes are shown to biologically interact with DISC1, it would have been interesting to investigate whether these genes demonstrate further combined effects that increase schizophrenia susceptibility within the DISC1 pathways. However due to the potentially large number of interactions in these pathways, our sample size remains too small for testing this meaningfully (6, 33). For the same reason, testing for interaction between the most significant Finnish DISC1 haplotype HEP3 (frequency = 0.088) and the variants recognized in the current study (frequencies ranging from 0.058 to 0.37) remains unreliable as only 0.51 % to 3.3 % of the studied individuals would carry combinations of these recognized variants. Yet, as the variants detected here are associated with schizophrenia independent of DISC1, it demonstrates that alterations in other components on the DISC1 pathway than just DISC1 itself may also influence schizophrenia etiology.

Emerging evidence supports a wider role for DISC1 in the development of various psychiatric and neurodevelopmental disorders with the latest observation being an association to the early age neurodevelopmental disorders autism and Asperger's syndrome (8) . Interestingly altered expression levels at PDE4B were recently reported within individuals affected with autism (72). PDE4B has as well been shown to associate with major depression (73). Further PDE4D has been reported to be associated with neuroticism, a psychological trait evidently highly related to major depression and anxiety (74). This would suggest that like DISC1, the other genes in the DISC1 pathways might play a general role in the development of neurodevelopmental disorders rather than being specific to schizophrenia, potentially being involved in a wider spectrum of psychiatric illness.

We have taken the novel approach of studying candidate genes for schizophrenia based on the known molecular interactions of a previously identified susceptibility gene. This "guided candidate gene approach" is theoretically attractive since it is plausible that variants in genes involved in the same molecular pathways can cause similar phenotypes. DISC1 is an excellent starting point on which to anchor such an effort. First it has been shown to be associated with schizophrenia both in our study sample and others and there is an abundance of independent, multimodal findings implying this gene in schizophrenia. DISC1 is known to function as a "hub" for many protein interactions acting along several pathways supporting this approach for this particular gene. Using this approach we observed significant association between schizophrenia and two memory related phosphodiesterase genes (PDE4B and PDE4D) as well as one neurodevelopmentally important peptidase gene (NDEL1). Combined with our previous observation of association with NDE1, this provides strong evidence supporting the DISC1 related pathways in the susceptibility of schizophrenia, and should inspire further research into these pathways.

ACKNOWLEDGEMENTS

The authors would like to acknowledge Sarah Whittall for help in producing the figures.

FINANCIAL DISCLOSURES

This work was funded in by The Centre of Excellence of the Academy of Finland and Biocentrum Helsinki Foundation (L. Peltonen). L. Tomppo is supported by Sigrid Juselius foundation. W. Hennah is a long term EMBO Research Fellow and was also supported by the Finnish Cultural Foundation Piippa Stiina Immonen grant. A. Loukola and A. Tuulio-Henriksson are supported by Academy of Finland post-doctoral fellowships. J. Suvisaari is supported by Academy of Finland research fellowship. L. Peltonen is on the board of Directors of Orion Pharma and Rocla. P. Lahermo, T. Partonen, J. Ekelund and J. Lönnqvist did not report any biomedical financial interests.

CONFLICT OF INTEREST STATEMENT

The authors declare they have no potential conflicts of interest.

SUPPLEMENTATY MATERIAL

Supplementary information is available at Biological Psychiatry Online.

REFERENCES

1. St Clair DM, Blackwood, D., Muir, W., Carothers, A., Walker, M., Spowart, G., Gosden, C., Evans, H.J. (1990): Association within a family of balanced autosomal translocation with major mental illness. *Lancet*.13-16.

2. Blackwood DH, Fordyce A, Walker MT, St Clair DM, Porteous DJ, Muir WJ (2001): Schizophrenia and affective disorders--cosegregation with a translocation at chromosome 1q42 that directly disrupts brain-expressed genes: clinical and P300 findings in a family. *Am J Hum Genet*. 69:428-433.

3. Ekelund J, Hovatta I, Parker A, Paunio T, Varilo T, Martin R, et al. (2001): Chromosome 1 loci in Finnish schizophrenia families. *Hum Mol Genet*. 10:1611-1617.

4. Ekelund J, Lichtermann D, Hovatta I, Ellonen P, Suvisaari J, Terwilliger JD, et al. (2000): Genome-wide scan for schizophrenia in the Finnish population: evidence for a locus on chromosome 7q22. *Hum Mol Genet*. 9:1049-1057.

5. Ekelund J, Hennah W, Hiekkalinna T, Parker A, Meyer J, Lonnqvist J, et al. (2004): Replication of 1q42 linkage in Finnish schizophrenia pedigrees. *Mol Psychiatry*. 9:1037-1041.

6. Hennah W, Varilo T, Kestila M, Paunio T, Arajarvi R, Haukka J, et al. (2003): Haplotype transmission analysis provides evidence of association for DISC1 to schizophrenia and suggests sex-dependent effects. *Hum Mol Genet*. 12:3151-3159.

7. Palo OM, Antila M, Silander K, Hennah W, Kilpinen H, Soronen P, et al. (2007): Association of distinct allelic haplotypes of DISC1 with psychotic and bipolar spectrum disorders and with underlying cognitive impairments. *Hum Mol Genet*. 16:3517-3528.

8. Kilpinen H, Ylisaukko-Oja T, Hennah W, Palo OM, Varilo T, Vanhala R, et al. (2008): Association of DISC1 with autism and Asperger syndrome. *Mol Psychiatry*. 13:187-196.

9. Cannon TD, Hennah W, van Erp TG, Thompson PM, Lonnqvist J, Huttunen M, et al. (2005): Association of DISC1/TRAX haplotypes with schizophrenia, reduced prefrontal gray matter, and impaired short- and long-term memory. *Arch Gen Psychiatry*. 62:1205-1213.

10. Hennah W, Tuulio-Henriksson A, Paunio T, Ekelund J, Varilo T, Partonen T, et al. (2005): A haplotype within the DISC1 gene is associated with visual memory functions in families with a high density of schizophrenia. *Mol Psychiatry*. 10:1097-1103.

11. Chen QY, Chen Q, Feng GY, Lindpaintner K, Wang LJ, Chen ZX, et al. (2007): Case-control association study of Disrupted-in-Schizophrenia-1 (DISC1) gene and schizophrenia in the Chinese population. *J Psychiatr Res*. 41:428-434.

12. DeRosse P, Hodgkinson CA, Lencz T, Burdick KE, Kane JM, Goldman D, et al. (2007): Disrupted in schizophrenia 1 genotype and positive symptoms in schizophrenia. *Biol Psychiatry*. 61:1208-1210.

13. Hashimoto R, Numakawa T, Ohnishi T, Kumamaru E, Yagasaki Y, Ishimoto T, et al. (2006): Impact of the DISC1 Ser704Cys polymorphism on risk for major depression, brain morphology and ERK signaling. *Hum Mol Genet*. 15:3024-3033.

14. Hodgkinson CA, Goldman D, Jaeger J, Persaud S, Kane JM, Lipsky RH, et al. (2004): Disrupted in schizophrenia 1 (DISC1): association with schizophrenia, schizoaffective disorder, and bipolar disorder. *Am J Hum Genet*. 75:862-872.

15. Kockelkorn TT, Arai M, Matsumoto H, Fukuda N, Yamada K, Minabe Y, et al. (2004): Association study of polymorphisms in the 5' upstream region of human DISC1 gene with schizophrenia. *Neurosci Lett*. 368:41-45.

16. Maeda K, Nwulia E, Chang J, Balkissoon R, Ishizuka K, Chen H, et al. (2006): Differential expression of disrupted-in-schizophrenia (DISC1) in bipolar disorder. *Biol Psychiatry*. 60:929-935.

17. Qu M, Tang F, Yue W, Ruan Y, Lu T, Liu Z, et al. (2007): Positive association of the Disrupted-in-Schizophrenia-1 gene (DISC1) with schizophrenia in the Chinese Han population. *Am J Med Genet B Neuropsychiatr Genet*. 144:266-270.

18. Thomson PA, Wray NR, Millar JK, Evans KL, Hellard SL, Condie A, et al. (2005): Association between the TRAX/DISC locus and both bipolar disorder and schizophrenia in the Scottish population. *Mol Psychiatry*. 10:657-668, 616.

19. Zhang F, Sarginson J, Crombie C, Walker N, St Clair D, Shaw D (2006): Genetic association between schizophrenia and the DISC1 gene in the Scottish population. *Am J Med Genet B Neuropsychiatr Genet*. 141:155-159.

20. Zhang X, Tochigi M, Ohashi J, Maeda K, Kato T, Okazaki Y, et al. (2005): Association study of the DISC1/TRAX locus with schizophrenia in a Japanese population. *Schizophr Res*. 79:175-180.

21. Thomson PA, Harris SE, Starr JM, Whalley LJ, Porteous DJ, Deary IJ (2005): Association between genotype at an exonic SNP in DISC1 and normal cognitive aging. *Neurosci Lett*. 389:41-45.

22. Liu YL, Fann CS, Liu CM, Chen WJ, Wu JY, Hung SI, et al. (2006): A single nucleotide polymorphism fine mapping study of chromosome 1q42.1 reveals the vulnerability genes for schizophrenia, GNPAT and DISC1: Association with impairment of sustained attention. *Biol Psychiatry*. 60:554-562.

23. Burdick KE, Hodgkinson CA, Szeszko PR, Lencz T, Ekholm JM, Kane JM, et al. (2005): DISC1 and neurocognitive function in schizophrenia. *Neuroreport*. 16:1399-1402.

24. Callicott JH, Straub RE, Pezawas L, Egan MF, Mattay VS, Hariri AR, et al. (2005): Variation in DISC1 affects hippocampal structure and function and increases risk for schizophrenia. *Proc Natl Acad Sci U S A*. 102:8627-8632.

25. Austin CP, Ky B, Ma L, Morris JA, Shughrue PJ (2004): Expression of Disrupted-In-Schizophrenia-1, a schizophrenia-associated gene, is prominent in the mouse hippocampus throughout brain development. *Neuroscience*. 124:3-10.

26. Miyoshi K, Honda A, Baba K, Taniguchi M, Oono K, Fujita T, et al. (2003): Disrupted-In-Schizophrenia 1, a candidate gene for schizophrenia, participates in neurite outgrowth. *Mol Psychiatry*. 8:685-694.

27. Schurov IL, Handford EJ, Brandon NJ, Whiting PJ (2004): Expression of disrupted in schizophrenia 1 (DISC1) protein in the adult and developing mouse brain indicates its role in neurodevelopment. *Mol Psychiatry*. 9:1100-1110.

28. Camargo LM, Collura V, Rain JC, Mizuguchi K, Hermjakob H, Kerrien S, et al. (2007): Disrupted in Schizophrenia 1 Interactome: evidence for the close connectivity of risk genes and a potential synaptic basis for schizophrenia. *Mol Psychiatry*. 12:74-86.

29. Millar JK, Christie S, Porteous DJ (2003): Yeast two-hybrid screens implicate DISC1 in brain development and function. *Biochem Biophys Res Commun*. 311:1019-1025.

30. Miyoshi K, Asanuma M, Miyazaki I, Diaz-Corrales FJ, Katayama T, Tohyama M, et al. (2004): DISC1 localizes to the centrosome by binding to kendrin. *Biochem Biophys Res Commun*. 317:1195-1199.

31. Morris JA, Kandpal G, Ma L, Austin CP (2003): DISC1 (Disrupted-In-Schizophrenia 1) is a centrosome-associated protein that interacts with MAP1A, MIPT3, ATF4/5 and NUDEL: regulation and loss of interaction with mutation. *Hum Mol Genet*. 12:1591-1608.

32. Ozeki Y, Tomoda T, Kleiderlein J, Kamiya A, Bord L, Fujii K, et al. (2003): Disrupted-in-Schizophrenia-1 (DISC-1): mutant truncation prevents binding to NudE-like (NUDEL) and inhibits neurite outgrowth. *Proc Natl Acad Sci U S A*. 100:289-294.

33. Hennah W, Tomppo L, Hiekkalinna T, Palo OM, Kilpinen H, Ekelund J, et al. (2007): Families with the risk allele of DISC1 reveal a link between schizophrenia and another component of the same molecular pathway, NDE1. *Hum Mol Genet*. 16:453-462.

34. Burdick KE, Kamiya A, Hodgkinson CA, Lencz T, DeRosse P, Ishizuka K, et al. (2008): Elucidating the relationship between DISC1, NDEL1 and NDE1 and the risk for schizophrenia: evidence of epistasis and competitive binding. *Hum Mol Genet*. 17:2462-2473.

35. Millar JK, Pickard BS, Mackie S, James R, Christie S, Buchanan SR, et al. (2005): DISC1 and PDE4B are interacting genetic factors in schizophrenia that regulate cAMP signaling. *Science*. 310:1187-1191.

36. Fatemi SH, King DP, Reutiman TJ, Folsom TD, Laurence JA, Lee S, et al. (2008): PDE4B polymorphisms and decreased PDE4B expression are associated with schizophrenia. *Schizophr Res*. 101:36-49.

37. Numata S, Ueno SI, Iga JI, Song H, Nakataki M, Tayoshi S, et al. (2008): Positive association of the PDE4B (phosphodiesterase 4B) gene with schizophrenia in the Japanese population. *J Psychiatr Res*.

38. Pickard BS, Thomson PA, Christoforou A, Evans KL, Morris SW, Porteous DJ, et al. (2007): The PDE4B gene confers sex-specific protection against schizophrenia. *Psychiatr Genet*. 17:129-133.

39. Yamada K, Nakamura K, Minabe Y, Iwayama-Shigeno Y, Takao H, Toyota T, et al. (2004): Association analysis of FEZ1 variants with schizophrenia in Japanese cohorts. *Biol Psychiatry*. 56:683-690.

40. Hennah W, Thomson P, Peltonen L, Porteous D (2006): Genes and Schizophrenia: Beyond Schizophrenia: The Role of DISC1 in Major Mental Illness. *Schizophr Bull*. 32:409-416.

41. Lichtermann D, Hovatta I, Terwilliger JD, Peltonen L, Lonnqvist J (1998): Concordance for sex and the pseudoautosomal gene hypothesis revisited: no evidence of increased sex concordance in a nationwide Finnish sample of siblings with paternally derived schizophrenia. *Am J Psychiatry*. 155:1365-1375.

42. (1994): American Psychiatric Association, Diagnostic and Statistcal Manual of Mental Disorders. Washington, DC: American Psychiatric Press.

43. Tienari P, Wynne LC, Laksy K, Moring J, Nieminen P, Sorri A, et al. (2003): Genetic boundaries of the schizophrenia spectrum: evidence from the Finnish Adoptive Family Study of Schizophrenia. *Am J Psychiatry*. 160:1587-1594.

44. Blackwood DH, Pickard BJ, Thomson PA, Evans KL, Porteous DJ, Muir WJ (2007): Are some genetic risk factors common to schizophrenia, bipolar disorder and depression? Evidence from DISC1, GRIK4 and NRG1. *Neurotox Res*. 11:73-83.

45. Shifman S, Bronstein M, Sternfeld M, Pisante A, Weizman A, Reznik I, et al. (2004): COMT: a common susceptibility gene in bipolar disorder and schizophrenia. *Am J Med Genet B Neuropsychiatr Genet*. 128:61-64.

46. Tuulio-Henriksson A, Haukka J, Partonen T, Varilo T, Paunio T, Ekelund J, et al. (2002): Heritability and number of quantitative trait loci of neurocognitive functions in families with schizophrenia. *Am J Med Genet*. 114:483-490.

47. Wechsler D (1987): Wechsler Memory Scale - Revised (WMS-R), Manual. Cleveland: Psychological Corporation, Habour Brace Jovanovich, Inc.

.

48. Delis DC, Kramer, J.H., Kaplan, E. and Ober, B.A. (1987): California Verbal Learning Test: Manual, Research Edition. San Antonio: Psychological Corporation, Harcourt Brace & Company

49. Wechsler D (1981): Wechsler Adult Inteligence Scale - Revised. Cleveland: Psychological Corporation, Harcourt Brace Jovanovich, Inc.

50. (2003): The International HapMap Project. *Nature*. 426:789-796.

51. Barrett JC, Fry B, Maller J, Daly MJ (2005): Haploview: analysis and visualization of LD and haplotype maps. *Bioinformatics*. 21:263-265.

52. Hedrick PW (1987): Gametic disequilibrium measures: proceed with caution. *Genetics*. 117:331-341.

53. Hinds DA, Stuve LL, Nilsen GB, Halperin E, Eskin E, Ballinger DG, et al. (2005): Whole-genome patterns of common DNA variation in three human populations. *Science*. 307:1072-1079.

54. Jurinke C, van den Boom D, Cantor CR, Koster H (2001): Automated genotyping using the DNA MassArray technology. *Methods Mol Biol*. 170:103-116.

55. O'Connell JR, Weeks DE (1998): PedCheck: a program for identification of genotype incompatibilities in linkage analysis. *Am J Hum Genet*. 63:259-266.

56. van den Oord EJ, Sullivan PF (2003): A framework for controlling false discovery rates and minimizing the amount of genotyping in the search for disease mutations. *Hum Hered*. 56:188-199.

57. Wen SH, Tzeng JY, Kao JT, Hsiao CK (2006): A two-stage design for multiple testing in large-scale association studies. *J Hum Genet*. 51:523-532.

58. Goring HH, Terwilliger JD (2000): Linkage analysis in the presence of errors IV: joint pseudomarker analysis of linkage and/or linkage disequilibrium on a mixture of pedigrees and singletons when the mode of inheritance cannot be accurately specified. *Am J Hum Genet*. 66:1310-1327.

59. Sobel E, Lange K (1996): Descent graphs in pedigree analysis: applications to haplotyping, location scores, and marker-sharing statistics. *Am J Hum Genet*. 58:1323-1337.

60. Abecasis GR, Cardon LR, Cookson WO (2000): A general test of association for quantitative traits in nuclear families. *Am J Hum Genet*. 66:279-292.

61. Abecasis GR, Cookson WO, Cardon LR (2000): Pedigree tests of transmission disequilibrium. *Eur J Hum Genet*. 8:545-551.

62. Lipska BK, Peters T, Hyde TM, Halim N, Horowitz C, Mitkus S, et al. (2006): Expression of DISC1 binding partners is reduced in schizophrenia and associated with DISC1 SNPs. *Hum Mol Genet*. 15:1245-1258.

63. Sasaki S, Mori D, Toyo-oka K, Chen A, Garrett-Beal L, Muramatsu M, et al. (2005): Complete loss of Ndel1 results in neuronal migration defects and early embryonic lethality. *Mol Cell Biol*. 25:7812-7827.

64. Brandon NJ, Handford EJ, Schurov I, Rain JC, Pelling M, Duran-Jimeniz B, et al. (2004): Disrupted in Schizophrenia 1 and Nudel form a neurodevelopmentally regulated protein complex: implications for schizophrenia and other major neurological disorders. *Mol Cell Neurosci*. 25:42-55.

65. Wynshaw-Boris A, Gambello MJ (2001): LIS1 and dynein motor function in neuronal migration and development. *Genes Dev*. 15:639-651.

66. Wedenoja J, Loukola A, Tuulio-Henriksson A, Paunio T, Ekelund J, Silander K, et al. (2007): Replication of linkage on chromosome 7q22 and association of the regional Reelin gene with working memory in schizophrenia families. *Mol Psychiatry*.

67. Schaar BT (2004): Cortical development deNUDEd. *Neuron*. 44:213-214.

68. Duan X, Chang JH, Ge S, Faulkner RL, Kim JY, Kitabatake Y, et al. (2007): Disrupted-In-Schizophrenia 1 regulates integration of newly generated neurons in the adult brain. *Cell*. 130:1146-1158.

69. Aleman A, Hijman R, de Haan EH, Kahn RS (1999): Memory impairment in schizophrenia: a meta-analysis. *Am J Psychiatry*. 156:1358-1366.

70. Davis RL, Cherry J, Dauwalder B, Han PL, Skoulakis E (1995): The cyclic AMP system and Drosophila learning. *Mol Cell Biochem*. 149-150:271-278.

71. Giorgi M, Modica A, Pompili A, Pacitti C, Gasbarri A (2004): The induction of cyclic nucleotide phosphodiesterase 4 gene (PDE4D) impairs memory in a water maze task. *Behav Brain Res*. 154:99-106.

72. Braun NN, Reutiman TJ, Lee S, Folsom TD, Fatemi SH (2007): Expression of phosphodiesterase 4 is altered in the brains of subjects with autism. *Neuroreport*. 18:1841-1844.

73. Numata S, Iga JI, Nakataki M, Tayoshi S, Taniguchi K, Sumitani S, et al. (2008): Gene expression and association analyses of the phosphodiesterase 4B (PDE4B) gene in major depressive disorder in the Japanese population. Am J Med Genet B Neuropsychiatr Genet.

74. Shifman S, Bhomra A, Smiley S, Wray NR, James MR, Martin NG, et al. (2008): A whole genome association study of neuroticism using DNA pooling. Mol Psychiatry. 13:302-312.

Legends to Figures

Figure 1

DISC1 binding partners included in the study with partners grouped by common functions.

Figure 2.

Schematics show the regions of interest on chromosomes 1 (a), 5 (b) and 17 (c), where PDE4B, PDE4D and NDEL1 are located respectively according to UCSC build 16 (released Jul. 2003). Genotyped SNPs are indicated with vertical lines. LD structure describes the haplotype blocks that were tested for association. For the SNPs displaying association and SNPs included in the associating haplotype blocks the rs-numbers are provided. Arrows above the schematics indicate direction from 5' to 3'.

Table 1. Summary of the results in the combined sample of the SNPs and haplotypes that passed replication in two stages.

Gene	Block	SNP	SNPs			Haplotypes				
			P	F(Affected)	F(Control)	Global P	Alleles	P	F(Affected)	F(Control)
NDEL1	1	rs17806986	0.0038	0.27	0.37	0.033	CGCG	0.0027	0.12	0.19
		rs1391768	nt	nt	nt					
		rs1391766	nt	nt	nt					
		rs3817003	nt	nt	nt					
PDE4B	4	rs4503327	nt	nt	nt	0.0060	CCC	0.029	0.28	0.21
		rs2503222	nt	nt	nt					
		rs6588186	nt	nt	nt		CTT	0.0022	0.022	0.058
PDE4B	5	rs10158178	nt	nt	nt	nt	nt			
		rs7412571	0.018	0.45	0.35					
		rs599235	nt	nt	nt					
		rs2069278	nt	nt	nt					
PDE4D	15	rs13190249	nt	nt	nt	0.0034	GGACA	0.00084	0.40	0.28
		rs1120303	0.021	0.13	0.19					
		rs921942	nt	nt	nt					
		rs10805515	nt	nt	nt					
		rs10514862	nt	nt	nt					

P-values (P) for the significant SNPs and haplotypes are provided with respective allele frequencies (F). SNPs and haplotypes displaying no evidence for association in stage 1 and stage 2 were not tested (nt).

Table 2. Examination of the differences between numbers of observations of the recognized variants and affected males and females

Gene	Block	SNP	P	F(Allele+)		F(Allele-)		Haplotype	P	F(Haplotype+)		F(Haplotype-)	
				M	F	M	F			M	F	M	F
NDEL1	1	rs17806986	0.57	0.28	0.29	0.72	0.71	CGCG	0.10	0.11	0.13	0.89	0.87
PDE4B	4	nt						CCC	0.227	0.29	0.28	0.71	0.72
								CTT	0.47	0.02	0.02	0.98	0.98
PDE4B	5	rs7412571	0.068	0.56	0.61	0.44	0.39	nt					
PDE4D	15	rs1120303	0.10	0.15	0.12	0.85	0.88	GGACA	0.10	0.39	0.42	0.61	0.58

Affected males and females where divided in groups based on the SNP and haplotype variants recognized. The testing for deviation in the haplotype and allelic distribution between males and females was performed using the chi squared test. Observations for associated SNP and haplotype are indicated for males (M) and females (F). F(Allele+) and F(Haplotype+) indicate frequencies of the associated variant meanwhile F(Allele-) and F(Haplotype-) indicate frequencies of other than the tested variant. The SNPs and haplotypes displaying no evidence for association in stage 1 or stage 2 were not tested (nt).

Figure
Click here to download high resolution image

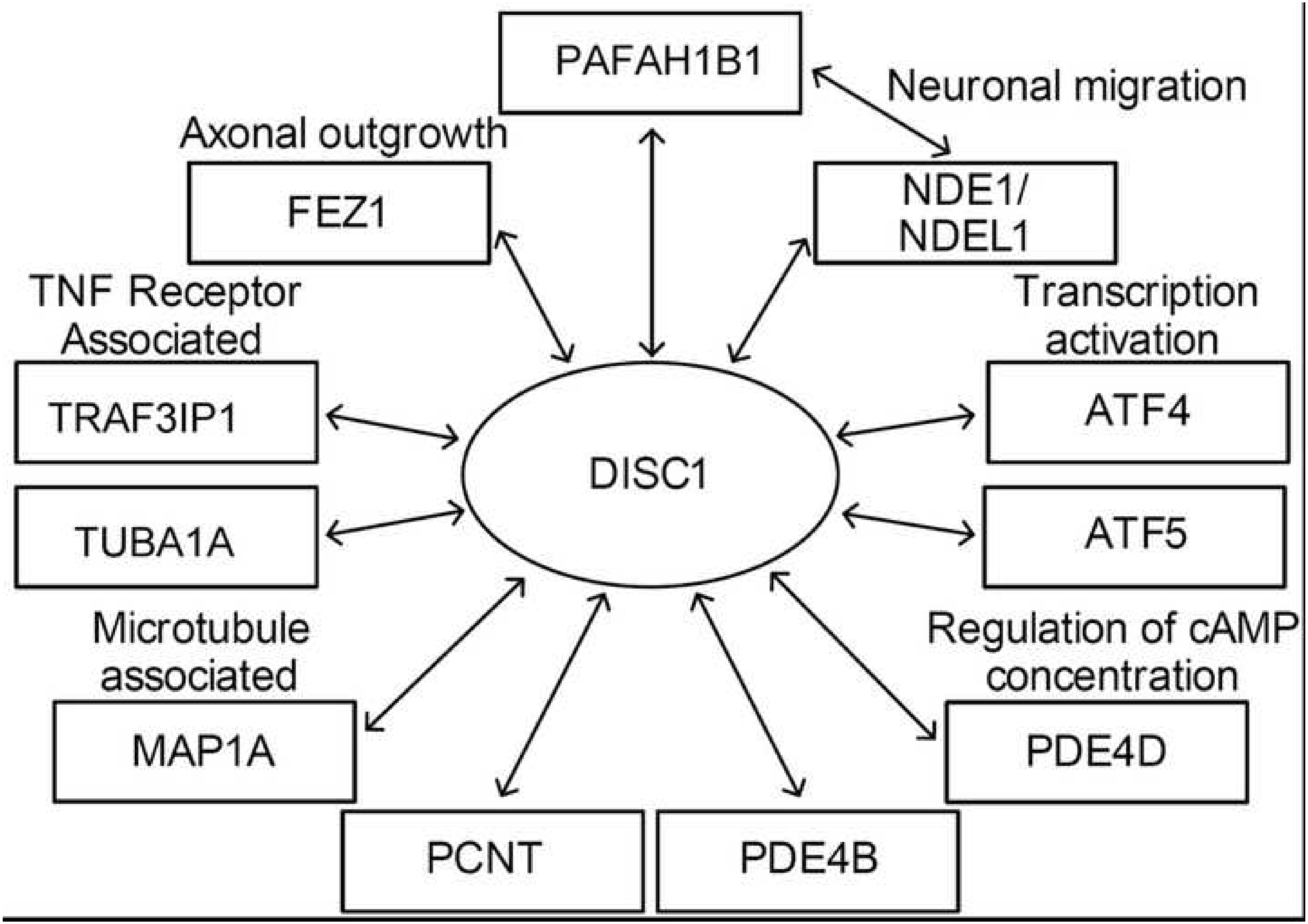

Figure
Click here to download high resolution image

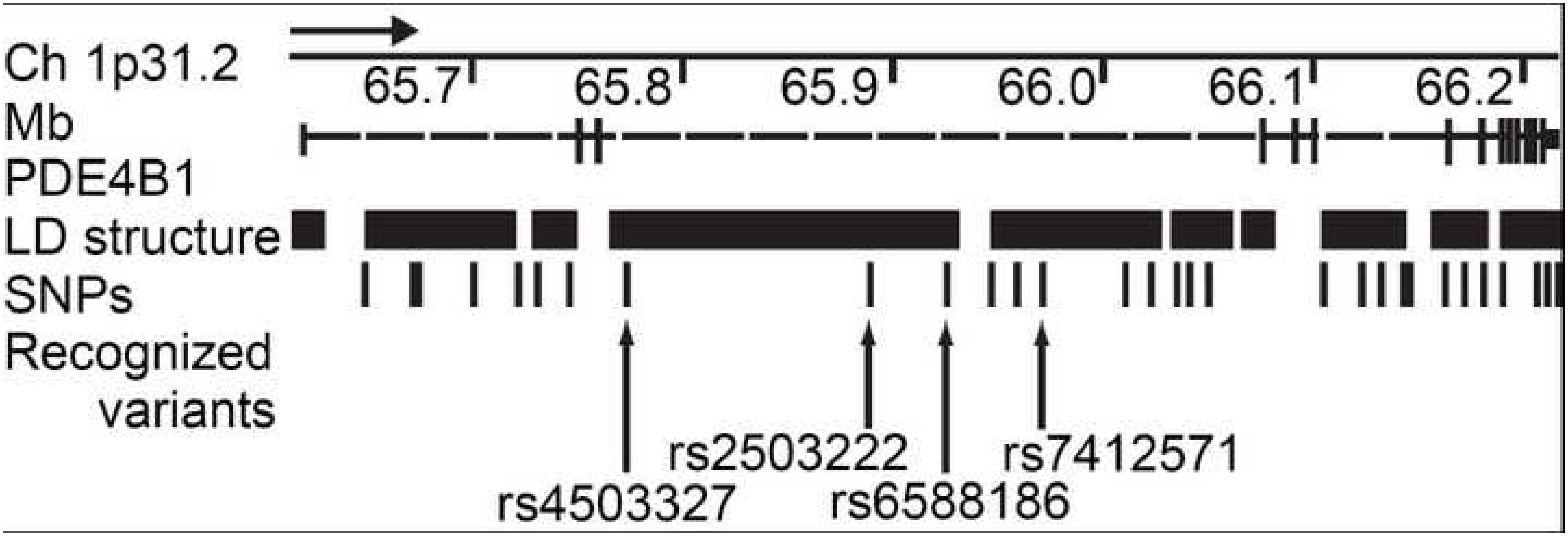

Figure
Click here to download high resolution image

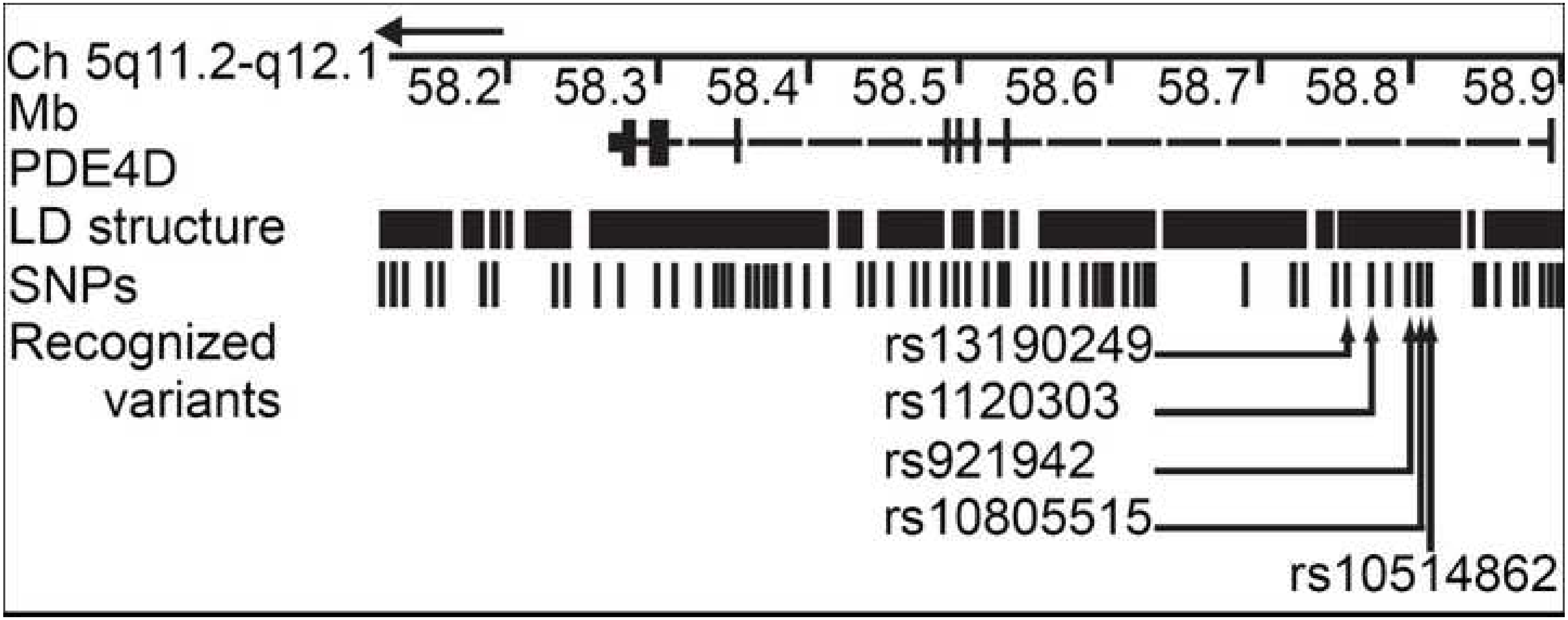

Figure
Click here to download high resolution image

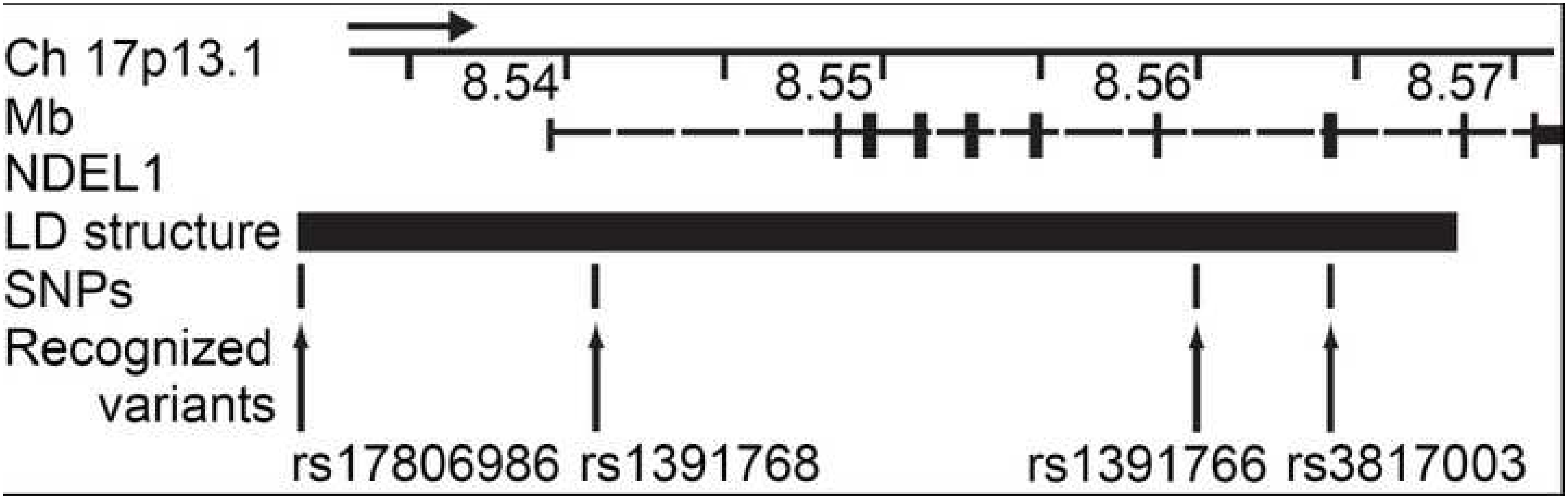

Supplementary Information Table 1. SNP association results for all the genes and SNPs.

Gene / rs	P-value		
	Stage1	Stage2	Combined
**ATF5**			
rs3826777	0.462		
rs8667	0.584		
rs1152230	0.327		
rs8647	0.406		
**FEZ1**			
rs1615640	0.655		
rs2241514	0.138		
rs597570	0.254		
rs2849222	0.439		
rs2155128	0.317		
**MAP1A**			
rs529611	0.164		
rs694985	0.064		
rs480108	0.115		
rs689797	0.136		
**NDEL1**			
rs17806986	0.022	0.003	0.004
rs1391768	0.003	0.480	
rs1391766	0.004	0.584	
rs3817003	0.003	0.403	
**PAFAH1B1**			
rs8068673	0.439		
rs9905797	0.386		
rs1266475	0.507		
rs2240730	0.178		
rs7212450	0.218		
**PCNT**			
rs13373028	0.624		
rs11701361	0.791		
rs2073382	1.000		
rs2839243	0.522		
rs3788265	0.362		
rs2839252	0.480		
rs915578	0.566		
rs743347	0.632		
**PDE4B**			
rs11208756	0.251		
rs4288570	0.090		
rs4384209	0.458		
rs4314863	0.597		
rs11208758	0.403		
rs4077431	0.188		
rs10493389	0.806		
rs2840677	0.420		
rs7547294	0.752		
rs4353064	0.920		
rs4329483	0.560		
rs4503327	0.099		
rs6683044	0.191		

rs2503222	0.026	0.157	
rs6588186	0.066		
rs10158178	0.052		
rs12404118	0.454		
rs7412571	0.028	0.048	0.018
rs599235	0.597		
rs2069278	0.149		
rs583018	0.281		
rs4655821	0.566		
rs566954	0.823		
rs502958	0.204		
rs522037	0.222		
rs599381	0.121		
rs782689	0.066		
rs524770	0.046	0.129	
rs1556805	0.076		
rs910693	0.325		
rs491190	0.142		
rs2144719	0.122		
rs783062	0.532		
rs783050	0.888		
**PDE4D**			
rs4700309	0.578		
rs1878198	0.729		
rs7728528	0.064		
rs1423368	0.718		
rs10514872	0.572		
rs1516436	0.260		
rs468321	0.841		
rs2936198	0.920		
rs4699929	0.042	0.273	
rs702514	0.740		
rs702524	0.584		
rs4700316	0.081		
rs1909294	0.330		
rs2968003	0.689		
rs2291851	0.104		
rs2279737	0.136		
rs1353747	0.671		
rs10514870	0.221		
rs7730070	0.442		
rs6867053	0.439		
rs12654005	0.462		
rs2968018	0.647		
rs2910641	0.025	0.371	
rs1115728	0.337		
rs9968728	0.281		
rs10035950	0.617		
rs1014317	0.354		
rs997421	0.058		
rs3901540	0.406		
rs1824788	0.454		
rs2409627	0.498		
rs1345792	0.212		

rs716908	0.015	1.000	
rs8180396	0.112		
rs2112957	0.888		
rs27172	0.841		
rs27727	0.663		
rs6867240	1.000		
rs425384	0.313		
rs6450512	0.590		
rs153968	0.647		
rs27548	0.080		
rs35305	1.000		
rs27723	0.170		
rs26708	0.186		
rs10491353	0.374		
rs27184	0.296		
rs4699941	0.841		
rs2014012	0.597		
rs1824159	0.073		
rs27171	0.480		
rs378869	0.484		
rs2547917	0.188		
rs1817248	0.107		
rs2081092	0.078		
rs13190249	0.006	0.439	
rs1120303	0.006	0.027	0.021
rs12514658	0.359		
rs921942	0.399		
rs10805515	0.146		
rs10514862	0.480		
rs1870077	0.597		
rs40216	0.841		
rs702543	1.000		
rs159196	0.365		
rs295936	0.740		
rs294498	0.351		
rs10514859	0.920		
rs295943	0.294		
**TRAF3IP1**			
rs4663873	0.271		
rs6706911	0.019	1.000	
rs5018862	0.458		
rs11679972	0.042	0.655	
rs821806	1.000		
rs821808	0.740		
rs524336	1.000		
**TUBA1A**			
rs1874908	0.467		
rs1039225	0.262		

Supplementary Information Table 2. Haplotype association results for all the tested haplotypes

	Haplotype + strand	FREQUENCIES Affected offspring				CHI p-value		
		Stage 1	Stage 2	Combined	Control	Stage 1	Stage 2	Combined
**ATF5**								
Block1	GAG	0.27			0.24	0.277		
	AAA	0.41			0.41	0.885		
	GGG	0.26			0.20	0.112		
**FEZ1**								
Block1	ACATC	0.06			0.04	0.234		
	ACTCC	0.13			0.09	0.111		
	ACATT	0.17			0.19	0.604		
	GCATC	0.15			0.12	0.313		
	GTATT	0.25			0.27	0.571		
	GCACC	0.10			0.10	0.901		
**MAP1A**								
Block1	TTC	0.77			0.82	0.125		
	CTT	0.12			0.08	0.093		
	CCT	0.09	0.05		0.04	0.042	0.613	
**NDEL1**								
Block1	CAGA	0.16			0.16	0.924		
	GGGG	0.42	0.39		0.34	0.038	0.165	
	GGCG	0.26			0.24	0.616		
	CGCG	0.12	0.12	0.12	0.19	0.007	0.005	0.003
**PAFAH1B1**								
Block1	tagged by SNP rs8068673							
Block2	CA	0.54	0.47		0.45	0.028	0.626	
	GG	0.41			0.46	0.144		
	CG	0.03			0.04	0.538		
**PCNT**								
Block1	TCTGTG	0.38			0.39	0.805		
	CTCTTG	0.05			0.06	0.429		
	CCTGTG	0.15			0.14	0.607		
	TTCTTG	0.18			0.12	0.074		
	TCTGCA	0.12			0.09	0.349		
	TTCGTG	0.09			0.09	0.756		
**PDE4B**								
Block2	GCAA	0.08			0.05	0.154		
	AAAA	0.15			0.17	0.440		
	GCTG	0.42			0.39	0.450		
	GCTA	0.07			0.06	0.657		
	GAAA	0.13			0.13	0.999		
Block3	AA	0.30			0.27	0.513		
	AG	0.52			0.55	0.454		
	CA	0.17			0.17	0.938		
Block4	TTT	0.05			0.09	0.061		
	TCC	0.56			0.55	0.975		
	CCC	0.29	0.28	0.28	0.21	0.029	0.048	0.029
	TCT	0.06			0.06	0.862		
	CTT	0.02	0.03	0.02	0.06	0.001	0.025	0.002

Block5	GGT	0.30	0.28	0.22	0.028	0.067
	AGC	0.35		0.37	0.510	
	AGT	0.07	0.09	0.11	0.049	0.318
	AAT	0.24		0.20	0.284	
Block6	TA	0.57		0.52	0.260	
	GA	0.23		0.28	0.175	
	GG	0.19		0.18	0.618	
Block8	TG	0.58		0.53	0.202	
	AG	0.25		0.25	0.795	
	AA	0.17		0.20	0.331	
Block9	CGT	0.51	0.45	0.41	0.027	0.408
	TAC	0.35		0.36	0.673	
	TAT	0.09		0.08	0.563	
	TGC	0.03		0.05	0.088	
Block10	TGGT	0.49		0.45	0.295	
	GACG	0.27		0.31	0.297	
	GGGT	0.11		0.12	0.664	
**PDE4D**						
Block1	GC	0.47		0.49	0.682	
	TG	0.16		0.17	0.775	
	TC	0.34		0.32	0.627	
Block2	tagged by SNP rs1516436					
Block3	tagged by SNP rs468321					
Block5	GG	0.31		0.38	0.077	
	GT	0.33	0.27	0.24	0.014	0.356
	AG	0.35		0.37	0.650	
Block6	GCA	0.56		0.50	0.150	
	ATA	0.28		0.26	0.669	
	GTA	0.07		0.09	0.305	
	ATG	0.06		0.08	0.339	
Block7	tagged by SNP rs9968728					
Block8	CCCT	0.16		0.15	0.724	
	GCTT	0.15		0.13	0.648	
	GTCT	0.17		0.17	0.947	
	GCTC	0.23		0.17	0.064	
	CCTC	0.15		0.16	0.875	
	CCTT	0.08		0.12	0.165	
Block9	AC	0.25		0.20	0.131	
	GC	0.49		0.57	0.061	
	GT	0.18		0.20	0.518	
	AT	0.08	0.04	0.04	0.032	0.839
Block10	CTCG	0.56		0.55	0.882	
	TCCT	0.22		0.17	0.096	
	TCCG	0.10	0.15	0.17	0.009	0.313
Block11	tagged by SNP rs 6450512					
Block12	TCGGC	0.20		0.18	0.406	
	TCCGC	0.04		0.07	0.212	
	GAGGT	0.13		0.09	0.108	
	GAGAC	0.09		0.05	0.118	
	TACGT	0.07		0.08	0.678	
	TCCGT	0.20		0.19	0.783	
	TAGGT	0.02	0.03	0.05	0.044	0.156

	GACGT	0.04			0.06	0.145		
Block13	CGG	0.23	0.30		0.32	0.010	0.550	
	CAG	0.13			0.11	0.490		
	TGG	0.39			0.38	0.761		
	CGC	0.22			0.17	0.090		
Block14	tagged by SNP rs2081092							
Block15	GGCTA	0.16			0.16	0.999		
	GGCCA	0.08			0.12	0.079		
	AGACA	0.01	0.03		0.06	0.000011	0.056	
	GTACA	0.04			0.07	0.093		
	GGCCG	0.10			0.10	0.717		
	GGACA	0.39	0.41	0.40	0.28	0.0059	0.001	0.001
	GTCTA	0.06			0.06	0.968		
	GGCTG	0.09			0.06	0.247		
Block16	tagged by SNP rs1870077							
Block17	ACCC	0.18			0.15	0.324		
	ACGT	0.05			0.08	0.070		
	GTCC	0.51			0.52	0.736		
	GCGC	0.09	0.06		0.04	0.026	0.228	
	GCCC	0.04			0.06	0.261		
	GCCT	0.05			0.06	0.285		
**TRAF3IP1**								
Block1	TTT	0.42	0.33		0.33	0.034	0.935	
	CTT	0.21			0.24	0.412		
	TTC	0.25			0.25	0.847		
	CGT	0.08	0.15		0.15	0.006	0.791	
**TUBA1A**								
Block1	TG	0.53			0.55	0.532		
	GA	0.40			0.33	0.079		
	GG	0.04			0.06	0.233		
	TA	0.03			0.06	0.130		

Supplementary Information Table 3. Details on SNPs successfully genotyped in the present study

Gene	rs number [a]	Chr [b]	LD block [c]	Location [d]	Minor/major allele + strand	Minor allele frequency founders [e]	Minor allele frequency HapMap [f]
ATF5	**rs3826777**	19	1	55123200	A/G	0.475	0.364
ATF5	**rs1152230**	19	1	55125315	G/A	0.283	0.262
ATF5	rs8667	19	1	55128183	A/G	0.495	0.350
ATF5	**rs8647**	19	1	55128792	A/G	0.487	0.250
FEZ1	**rs1615640**	11	1	124845728	C/G	0.411	0.292
FEZ1	**rs2241514**	11	1	124863678	T/C	0.262	0.500
FEZ1	**rs597570**	11	1	124889124	T/A	0.227	0.167
FEZ1	**rs2849222**	11	1	124891141	C/T	0.339	0.233
FEZ1	**rs2155128**	11	1	124919725	T/C	0.466	0.357
MAP1A	**rs529611**	15	1	41522044	C/T	0.195	0.317
MAP1A	**rs694985**	15	1	41529443	C/T	0.078	0.067
MAP1A	rs480108	15	1	41533462	C/T	0.191	0.314
MAP1A	**rs689797**	15	1	41542613	T/C	0.190	0.289
NDEL1	**rs17806986**	17	1	8530610	C/G	0.292	0.500
NDEL1	**rs1391768**	17	1	8541432	G/A	0.455	0.408
NDEL1	**rs1391766**	17	1	8560069	C/G	0.446	0.408
NDEL1	**rs3817003**	17	1	8564190	G/A	0.448	0.408
PAFAH1B1	**rs8068673**	17	1	2708977	C/T	0.288	0.300
PAFAH1B1	rs9905797	17	1	2715952	T/C	0.289	0.300
PAFAH1B1	rs1266475	17	1	2741911	C/T	0.302	0.317
PAFAH1B1	**rs7212450**	17	2	2798531	G/C	0.458	0.439
PAFAH1B1	**rs2240730**	17	2	2800809	G/A	0.464	0.458
PCNT	**rs13373028**	21	1	46618576	C/T	0.205	0.314
PCNT	**rs11701361**	21	1	46620679	T/C	0.320	0.254
PCNT	**rs2073382**	21	1	46674500	C/T	0.322	0.381
PCNT	**rs2839243**	21	1	46687045	T/G	0.227	0.314
PCNT	rs3788265	21	1	46689991	C/T	0.022	0.161
PCNT	**rs2839252**	21	1	46700125	C/T	0.104	0.161
PCNT	**rs915578**	21	1	46702962	A/G	0.103	0.110
PCNT	rs743347	21	1	46720658	T/C	0.013	0.263
PDE4B	rs11208756	1		65639228	C/T	0.048	0.108
PDE4B	rs4288570	1		65646902	C/T	0.363	0.283
PDE4B	rs4384209	1		65649042	A/G	0.249	0.175
PDE4B	**rs11208758**	1	2	65657117	A/G	0.222	0.129
PDE4B	**rs4077431**	1	2	65679309	A/C	0.421	0.456
PDE4B	rs10493389	1	2	65680782	C/T	0.137	0.092
PDE4B	**rs2840677**	1	2	65703794	A/T	0.473	0.492
PDE4B	**rs7547294**	1	2	65721652	A/G	0.465	0.475
PDE4B	**rs4353064**	1	3	65727592	C/A	0.175	0.158
PDE4B	**rs4329483**	1	3	65743921	A/G	0.483	0.474
PDE4B	**rs4503327**	1	4	65776724	C/T	0.326	0.350
PDE4B	**rs2503222**	1	4	65888614	T/C	0.117	0.100
PDE4B	**rs6588186**	1	4	65916926	T/C	0.172	0.208
PDE4B	**rs10158178**	1	5	65929090	G/A	0.367	0.398
PDE4B	rs12404118	1	5	65943170	A/G	0.034	0.059
PDE4B	rs7412571	1	5	65954374	T/C	0.431	0.450
PDE4B	**rs599235**	1	5	65996290	A/G	0.260	0.267
PDE4B	**rs2069278**	1	5	66005288	C/T	0.373	0.314
PDE4B	**rs583018**	1	6	66016103	G/T	0.435	0.450
PDE4B	**rs4655821**	1	6	66019964	G/A	0.212	0.250

PDE4B	rs566954	1	6	66030772	C/G	0.184	0.185
PDE4B	**rs502958**	1	8	66101099	A/T	0.393	0.400
PDE4B	rs522037	1	8	66121818	G/C	0.398	0.392
PDE4B	**rs599381**	1	8	66130477	A/G	0.178	0.233
PDE4B	rs782689	1	8	66134869	G/C	0.065	0.058
PDE4B	rs524770	1	8	66136139	A/G	0.341	0.350
PDE4B	**rs1556805**	1	9	66154596	T/C	0.487	0.458
PDE4B	**rs910693**	1	9	66166165	A/G	0.464	0.408
PDE4B	**rs491190**	1	9	66180574	C/T	0.403	0.358
PDE4B	**rs2144719**	1	10	66193051	G/T	0.455	0.373
PDE4B	**rs783062**	1	10	66216421	A/G	0.326	0.233
PDE4B	**rs783061**	1	10	66216459	G/C	0.376	0.271
PDE4B	**rs783050**	1	10	66219365	G/T	0.382	0.275
PDE4B	rs1999856	1	10	66226941	C/A	0.331	0.167
PDE4D	rs4700309	5	1	58111107	C/T	0.435	0.425
PDE4D	**rs1878198**	5	1	58111447	A/T	0.487	0.458
PDE4D	rs7728528	5	1	58122180	G/T	0.079	0.076
PDE4D	rs1423368	5	1	58130991	T/C	0.488	0.483
PDE4D	**rs10514872**	5	1	58146926	G/C	0.191	0.267
PDE4D	**rs1516436**	5	2	58187140	C/G	0.310	0.342
PDE4D	**rs468321**	5	3	58191750	G/A	0.253	0.233
PDE4D	**rs2936198**	5	5	58235895	A/G	0.418	0.400
PDE4D	**rs4699929**	5	5	58241784	T/G	0.289	0.208
PDE4D	**rs702514**	5	6	58256566	A/G	0.375	0.367
PDE4D	**rs702524**	5	6	58275454	T/C	0.405	0.483
PDE4D	rs4700316	5	6	58310576	C/G	0.285	0.153
PDE4D	rs1909294	5	6	58322304	A/G	0.169	0.117
PDE4D	rs2968003	5	6	58336695	T/C	0.495	0.467
PDE4D	rs2291851	5	6	58347697	T/C	0.134	0.050
PDE4D	rs2279737	5	6	58350530	G/A	0.284	0.298
PDE4D	rs1353747	5	6	58353366	G/T	0.054	0.108
PDE4D	**rs10514870**	5	6	58367213	G/A	0.077	0.102
PDE4D	rs7730070	5	6	58373461	G/C	0.375	0.317
PDE4D	rs6867053	5	6	58381430	C/G	0.456	0.383
PDE4D	rs12654005	5	6	58386723	G/T	0.379	0.342
PDE4D	rs2968018	5	6	58401667	T/C	0.273	0.192
PDE4D	rs2910641	5	6	58412133	T/C	0.232	0.208
PDE4D	rs1115728	5	6	58423910	C/T	0.316	0.388
PDE4D	**rs9968728**	5	7	58434866	C/T	0.104	0.125
PDE4D	rs10035950	5		58438895	G/T	0.115	0.161
PDE4D	**rs1014317**	5	8	58445466	C/G	0.423	0.408
PDE4D	rs997421	5	8	58445527	T/C	0.224	0.085
PDE4D	**rs3901540**	5	8	58445618	T/C	0.216	0.275
PDE4D	**rs1824788**	5	8	58456143	C/T	0.381	0.425
PDE4D	rs2409627	5	8	58460207	C/T	0.159	0.178
PDE4D	rs1345792	5	8	58469916	T/C	0.489	0.400
PDE4D	**rs716908**	5	8	58485377	C/T	0.381	0.267
PDE4D	**rs8180396**	5	9	58497645	A/G	0.294	0.317
PDE4D	**rs2112957**	5	9	58507291	T/C	0.243	0.078
PDE4D	**rs27172**	5	10	58516017	T/C	0.404	0.467
PDE4D	**rs27727**	5	10	58516267	C/T	0.373	0.400
PDE4D	**rs6867240**	5	10	58524423	G/C	0.037	0.067
PDE4D	**rs425384**	5	10	58526211	T/G	0.211	0.142
PDE4D	**rs6450512**	5	11	58527386	A/G	0.414	0.458

PDE4D	rs153968	5		58538495	C/A	0.286	0.283
PDE4D	**rs27548**	5	12	58542956	G/T	0.382	0.267
PDE4D	**rs35305**	5	12	58564116	A/C	0.463	0.390
PDE4D	rs27723	5	12	58580117	G/A	0.331	0.203
PDE4D	**rs26708**	5	12	58586221	C/G	0.446	0.425
PDE4D	**rs10491353**	5	12	58591018	A/G	0.158	0.108
PDE4D	**rs27184**	5	12	58591208	C/T	0.453	0.408
PDE4D	rs4699941	5	12	58616079	T/C	0.352	0.292
PDE4D	rs2014012	5	12	58628273	T/A	0.307	0.308
PDE4D	rs1824159	5	12	58634529	T/C	0.079	0.100
PDE4D	rs27171	5	12	58634774	G/A	0.250	0.283
PDE4D	**rs378869**	5	13	58704590	T/C	0.405	0.400
PDE4D	**rs2547917**	5	13	58729565	A/G	0.164	0.192
PDE4D	**rs1817248**	5	13	58733188	C/G	0.187	0.178
PDE4D	**rs2081092**	5	14	58746170	G/A	0.110	0.092
PDE4D	**rs13190249**	5	15	58748546	A/G	0.049	0.095
PDE4D	**rs1120303**	5	15	58765816	T/G	0.149	0.108
PDE4D	rs12514658	5	15	58790846	T/G	0.185	0.200
PDE4D	**rs921942**	5	15	58803742	A/C	0.474	0.458
PDE4D	**rs10805515**	5	15	58809603	T/C	0.319	0.342
PDE4D	**rs10514862**	5	15	58813457	G/A	0.192	0.125
PDE4D	**rs1870077**	5	16	58850737	A/G	0.231	0.283
PDE4D	**rs40216**	5	17	58852835	A/G	0.281	0.367
PDE4D	**rs702543**	5	17	58858659	C/T	0.458	0.492
PDE4D	**rs159196**	5	17	58874959	G/C	0.209	0.207
PDE4D	rs295936	5	17	58881742	C/T	0.040	0.058
PDE4D	**rs294498**	5	17	58892835	T/C	0.173	0.250
PDE4D	rs10514859	5	17	58895707	A/C	0.074	0.092
PDE4D	rs295943	5	17	58902758	T/C	0.096	0.158
TRAF3IP1	**rs4663873**	2	1	239511954	C/T	0.384	0.475
TRAF3IP1	**rs6706911**	2	1	239516604	T/G	0.158	0.342
TRAF3IP1	**rs5018862**	2	1	239530498	C/T	0.261	0.225
TRAF3IP1	rs821806	2	1	239541325	G/A	0.274	0.350
TRAF3IP1	rs11679972	2	1	239541325	A/T	0.153	0.292
TRAF3IP1	rs821808	2	1	239549075	A/G	0.270	0.283
TRAF3IP1	rs524336	2	1	239587008	A/T	0.271	0.350
TUBA1A	**rs1039225**	12	1	47868959	G/T	0.391	0.290
TUBA1A	**rs1874908**	12	1	47870297	A/G	0.401	0.330

[a)] Tagging SNPs are indicated with bold
[b)] Chromosome
[c)] For SNPs located outside LD blocks, block number is blank
[d)] Location of the SNP according to UCSC database Jul 2003 build (hg16)
[e)] Minor allele frequency in founders in our combined study sample
[f)] Minor allele frequency according to HapMap CEU population

Tomppo et al (manuscript number PBS-D-08_01306) studied 11 genes involved in the "DISC1 pathway" in Finnish families ascertained for schizophrenia. They identified SNPs and haplotypes in PDE4B, PDE4D and NDEL1 that displayed evidence for association to schizophrenia.

This piece of the submission is being sent via mail.
